# Complete Chloroplast Genomes of 9 *Impatiens* Species: Genome Structure, Comparative Analysis, and Phylogenetic Relationships

**DOI:** 10.3390/ijms26020536

**Published:** 2025-01-10

**Authors:** Hui Ma, Zhiqiang Liu, Wenxiang Lan, Mengqing Yang, Qing Mo, Xi Huang, Peiqing Wu, Haiquan Huang, Meijuan Huang

**Affiliations:** College of Landscape Architecture and Horticulture Sciences, Southwest Research Center for Engineering Technology of Landscape Architecture (State Forestry and Grassland Administration), Yunnan Engineering Research Center for Functional Flower Resources and Industrialization, Research and Development Center of Landscape Plants and Horticulture Flowers, Southwest Forestry University, Kunming 650224, China; mahui04179@swfu.edu.cn (H.M.); lzq11147890@swfu.edu.cn (Z.L.); lanwenxiang@swfu.edu.cn (W.L.); yangmq@swfu.edu.cn (M.Y.); moqing@swfu.edu.cn (Q.M.); xihuang@swfu.edu.cn (X.H.); wupeiqing@swfu.edu.cn (P.W.)

**Keywords:** *Impatiens*, chloroplast genome, comparative genomics, single-nucleotide polymorphisms, selective pressure analysis, phylogenetic analysis

## Abstract

*Impatiens* is a genus of functional herbaceous plants in the Balsaminaceae, which are not only of great ornamental value and one of the world’s top three flower bedding plants but also have a wide range of medicinal and edible uses. Currently, the taxonomy and phylogenetic relationships of *Impatiens* species are still controversial. In order to better understand their chloroplast properties and phylogenetic evolution, nine *Impatiens* plants (*Impatiens repens*, *Impatiens rectirostrata*, *Impatiens baishaensis*, *Impatiens rostellata*, *Impatiens faberi*, *Impatiens oxyanthera*, *Impatiens tienchuanensis*, *Impatiens blepharosepala*, *Impatiens distracta*) were sequenced, and their complete chloroplast genomes were analysed. Nine species of *Impatiens* chloroplast genomes ranged in length from 150,810 bp (*I. rectirostrata*) to 152,345 bp (*I. blepharosepala*). The chloroplast genomes were all typical circular DNA molecules, and the GC content in each region was consistent with the published chloroplast genomes of *Impatiens* plants. The results showed that the seven mutational hotspots (*trnL-UAG*, *ndhG*, *ycf1*, *ccsA*, *rrn23*, *trnA-UGC*, and *ycf2*) could be used as supporting data for further analyses of the phylogenetic tree and species identification. In addition, the results of the phylogenetic tree support that Balsaminaceae is a monophyletic taxon, and that *Hydrocera triflora* is at the base of the branch, is the original species of Balsaminaceae, and is in a sister group relationship with *Impatiens* species. The results of this paper enrich the data of *Impatiens* chloroplast genomes, and the availability of these chloroplast genomes will provide rich genetic information for species identification, thus enhancing the taxonomic accuracy and phylogenetic resolution of *Impatiens*, and further promoting the investigation and rational use of *Impatiens* plant resources.

## 1. Introduction

Balsaminaceae comprises two genera, *Impatiens* and *Hydrocera* [1]. *Impatiens* is a large genus of Balsaminaceae, which is an annual or perennial herbaceous plant [2] that grows in altitudes ranging from 800 to 5000 metres [3], with five major centres of plant diversity distribution in tropical Africa, Madagascar, southern India and Sri Lanka, the Eastern Himalayas, and Southeast Asia [4,5]; the majority of *Impatiens* plants are intolerant of drought and direct sunlight [6]. New species of the genus have been discovered in recent years, and its species number has gradually increased [7,8]. *Impatiens* is very rich in China [9]; it is mainly distributed in the mountainous regions of southwestern and northwestern China, especially in Yunnan, Sichuan, Guizhou, and Tibet, where it is the most abundant species. *Impatiens* has a beautifully structured corolla with high ornamental value, and some *Impatiens* plants also have important medicinal functions and a wide range of folk clinical applications [10]. Modern pharmacological studies have shown that *Impatiens* possesses multiple biological activities, such as anti-cancer, antibacterial, pain relief, and anti-inflammatory [11]. The 2020 edition of the Pharmacopoeia of the People’s Republic of China includes Fenugreek seed acute as a standard medicinal plant [12]. In addition, many *Impatiens* species, such as *Impatiens hawkeri*, *Impatiens walleriana*, and *Impatiens balsamina*, are cultivated for ornamental purposes in many regions of the world and are also utilised as medicinal and food plants.

From a taxonomic point of view, Balsaminaceae plants are widely known for their taxonomic difficulties [13]. Their semi-polypody stems and fleshy leaf characteristics make obtaining dried and well-preserved plant specimens a difficult task. The flowers are extremely delicate, and during drying, most of them fold and aggregate, resulting in an exceptionally tedious separation and reconstruction of the parts [14]. *Impatiens* classification has traditionally been divided into species groups based on morphological and geographic characteristics (e.g., habit, sepal, and petal shape), and more recently, with the improvement of molecular taxonomy, the classification of *Impatiens* has been based on the plastid protein-coding genes *matK*, *rbcL*, and *trnK,* as well as the intergenic regions, *atpB-rbcL* and *trnL-trnF* [15,16]. *Impatiens* was classified into two subgen. According to Yu [17], the first subgen., Clavicarpa, is characterised mainly by racemes with more than five flowers, four fully developed lateral sepals, trichobezoar sprouting hole, four carpels, fusiform fruits, one ovule per carpel, and ovules with a simple reticulate ornamentation of the ellipsoidal seeds; the second subgen., *Impatiens* is characterised by having four pollen, five carpels, and more than two ovules per carpel. Some of the characters of the first subgen. also apply to some of the species of the second subgen. The subgenus *Impatiens* is divided further into seven sections: sect. *Semeiocardium*, sect. *Tuberosae*, sect. *Racemosae*, sect. *Impatiens*, sect. *Scorpioidae*, sect. *Fasciculatae*, and sect. *Uniflorae*. To date, many approaches have been proposed for the classification of *Impatiens*, some focusing on geography, some focusing on micromorphology, and hardly any one fixed approach. Therefore, further molecular and physiological refinements are needed for the classification of *Impatiens*.

Chloroplasts are a large energy conversion factory in eukaryotic cells, a kind of organelle in green higher plant cells, which can carry out semi-autonomous replication and photosynthesis, through which light energy can be converted into carbohydrates available to human beings [18], and therefore, chloroplasts are the crucial material basis for plant growth and development and human survival [19]. The chloroplast genome is mostly patrilineally inherited in gymnosperms and matrilineally inherited in angiosperms [20], and a few plants may be biparental [21]. The sequence structure and base composition of the chloroplast genome show a high degree of conservation, and compared with the nuclear genome, its nucleotide sequence has a low degree of variation. However, when compared with the mitochondrial genome, the chloroplast genome shows a relatively high level of nucleotide variation [22]. Chloroplast genomes of higher plants generally show a typical tetrameric structure, which consists of one Large Single Copy Region (LSC), one Small Single Copy Region (SSC), and two symmetrically distributed Inverted Repeat Regions (IRs), further subdivided into IRa and IRb [23]. This genomic architecture exists in the form of its ring covalent closure, reflecting the semi-autonomous nature of the chloroplast genome. The genetic information it encodes mainly contributes to the chloroplast’s own biosynthetic pathways, gene expression regulatory mechanisms, and especially critical photosynthetic processes, functions that are essential for maintaining plant life activities and energy conversion [24]. Currently, the chloroplast genome has become a key molecular tool and technology for plant germplasm identification, diversity analysis, phylogenetic studies, and species evolution [25]. Recent studies have shown that variation in the chloroplast genome provides important clues to unravelling developmental relationships in multilevel taxonomic systems, especially in groups of organisms with high taxonomic complexity [26,27]. To date, chloroplast genome analyses have extensively covered the major species of Gramineae [28], yet there are fewer relevant studies in Balsaminaceae plants. Morphological variation in *Impatiens* is extremely complex, resulting in significant differences in morphological characteristics between specimens and living plants, and is considered the most difficult taxon to study in angiosperm taxonomy [29].

In this study, the chloroplast genomes of nine *Impatiens* species were assembled by sequencing, and the genomes were analysed in depth; these samples are from China and Sri Lanka. These nine species were chosen mainly because their taxonomic status and affinities are controversial and not fully determined, and this uncertainty provides room for exploration. The research objectives of this paper are (1) to present the complete chloroplast genome sequences of nine *Impatiens* species; (2) to compare the chloroplast genome structures of *Impatiens*; (3) to examine SSR and repetitive sequence variation in the chloroplast genomes of the nine *Impatiens* species; (4) to compare the genomic differences and to identify regions of high mutation and mutant genes; and (5) to use chloroplast whole genomes to infer and test phylogenetic relationships among the Balsaminaceae and to improve understanding of the evolutionary and systematic position of *Impatiens* within the Balsaminaceae.

## 2. Results

### 2.1. General Features of the Impatiens Chloroplast Genome

**Figure 1:**
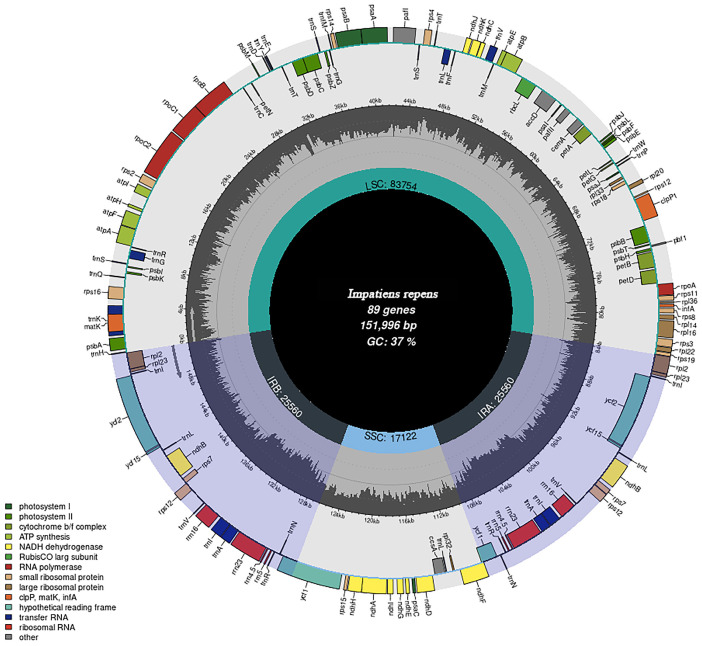
Gene map of the *I. repens* chloroplast genome. Genes transcribed clockwise are shown on the outside of the outer circle, while genes transcribed counterclockwise are located inside the inner circle. Genes with different functional groups are distinguished by colour coding. The positions of the long single-copy (LSC), short single-copy (SSC), and inverted repeat regions are shown in the inner circles.

By using Illumina MiSeq (Illumina Corporation, San Diego, CA, USA) sequencing, the reads were filtered, and the data were analysed to obtain that the length of the chloroplast genomes of the nine *Impatiens* species ranged from 150,810 bp (*I. rectirostrata*) to 152,345 bp (*I. blepharosepala*) (Table 1). The chloroplast genomes of all nine *Impatiens* species in this study were typical of circular DNA molecules, and the GC content in each region matches the published chloroplast genome of *Impatiens* plants (Figure 1 and Figures S1–S8). Like most angiosperms, the *Impatiens* chloroplast genome also exhibits a typical four-part structure, with the IR region ranging from 25,560 bp (*I. repens*) to 25,834 bp (*I. blepharosepala*) being complemented by the LSC region of 8020 bp (*I. rectirostrata*) to 83,754 bp (*I. repens*) and SSC region 17,122 bp (*I. repens*) to 17,616 bp (*I. rectirostrata*). The GC content in the chloroplast genomes of all nine *Impatiens* species was 37% (Table 1). And the GC content was distributed differently among the regions, with the GC content in the IR region being higher than that in the LSC region, which in turn was higher than that in the SSC region (Table 1). The number of genes in *I. repens*, *I. rectirostrata*, *I. rostellata,* and *I. distracta* was 147, 165, 147, and 166, of which 94, 104, 105, 94, and 105 were protein coding genes, respectively, and 12 rRNA genes in all of them, 41 tRNA genes in *I. repens* and *I. rostellata*, and 49 genes in the rest of the 2 species; *I. baishaensis*, *I. faberi*, *I. oxyanthera*, *I. tienchuanensis,* and *I. blepharosepala* had similar numbers of genes, 132, 133, 130, 130, and 132, respectively, of which 87, 88, 85, 85, and 87 were protein-coding genes, 8 were rRNA genes, and 37 were tRNA genes, respectively. The proteins encoded by these genes are involved in photosynthesis, self-replication, and others, among which unknown proteins are also present (Table 2 and Tables S1–S8). Of these, nineteen were intron-containing genes, and sixteen contained an intron (*ndhA*, *ndhB*, *petB*, *petD*, *atpF*, *rpl16*, *rpl2*, *rps16*, *rpoC1*, *trnA-UGC*, *trnG-GCC*, *trnG-UCC*, *trnI-GAU*, *trnK-UUU*, *trnL-UAA,* and *trnV-UAC*), and three contained two introns (*rps12*, *clpP1,* and *pafI*).

### 2.2. Codon Preference Analysis

**Figure 2:**
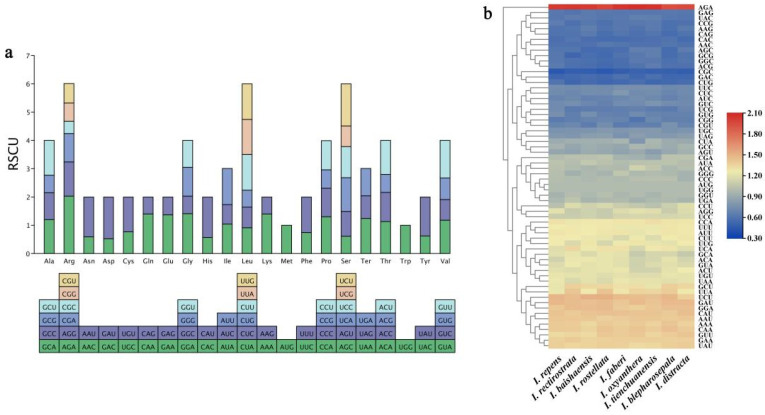
Relative synonymous codon usage (RSCU) in *Impatiens* plants. (**a**) Stacked bar chart of the relative synonymous codon usage (RSCU) in Tetrastigma plants, taking *I. repens* as an example; (**b**) heatmap displaying RSCU values.

The CDS sequences of the chloroplast genomes of nine *Impatiens* species were extracted for codon preference analysis (Figure 2). Sixty-four codons were used in all nine *Impatiens* plants, encoding 21 amino acids, and the protein-coding genes coded for codons 50,270 (*I. rectirostrata*) to 50,781 (*I. blepharosepala*). Among them, Leu (L), Arg (R), and Ser (S) had the highest number of codons, all amounting to six; Met (M) and Trp (W) each had only 1 codon, AUG and UGG, respectively, which were the least abundant. The codon with the highest number of occurrences was UUU encoding Phe (F) with 2310 (*I. rectirostrata*) to 2397 (*I. distracta*) codons; GCG encoding Ala (A) was the codon with the lowest number of codons for the 9 *Impatiens* species, with numbers ranging from 206 (*I. rectirostrata*) to 232 (*I. faberi*). The termination codons for all nine *Impatiens* species were more favourable to UAA, with RSCU values ranging from 1.16 (*I. rectirostrata*) to 1.24 (*I. repens* and *I. faberi*) (Tables S9–S17).

Codon types with RSCU values ≥ 1.00 were found in 32 (*I. faberi*) to 36 (*I. rectirostrata*) of the nine *Impatiens* plants, with a high number of occurrences and a preponderance of codons ending in A/U and only a few in C/G, a phenomenon similar to that of the chloroplast genomes of most angiosperms (Tables S9–S17).

### 2.3. Repeat Sequences and SSR Analysis

**Figure 3:**
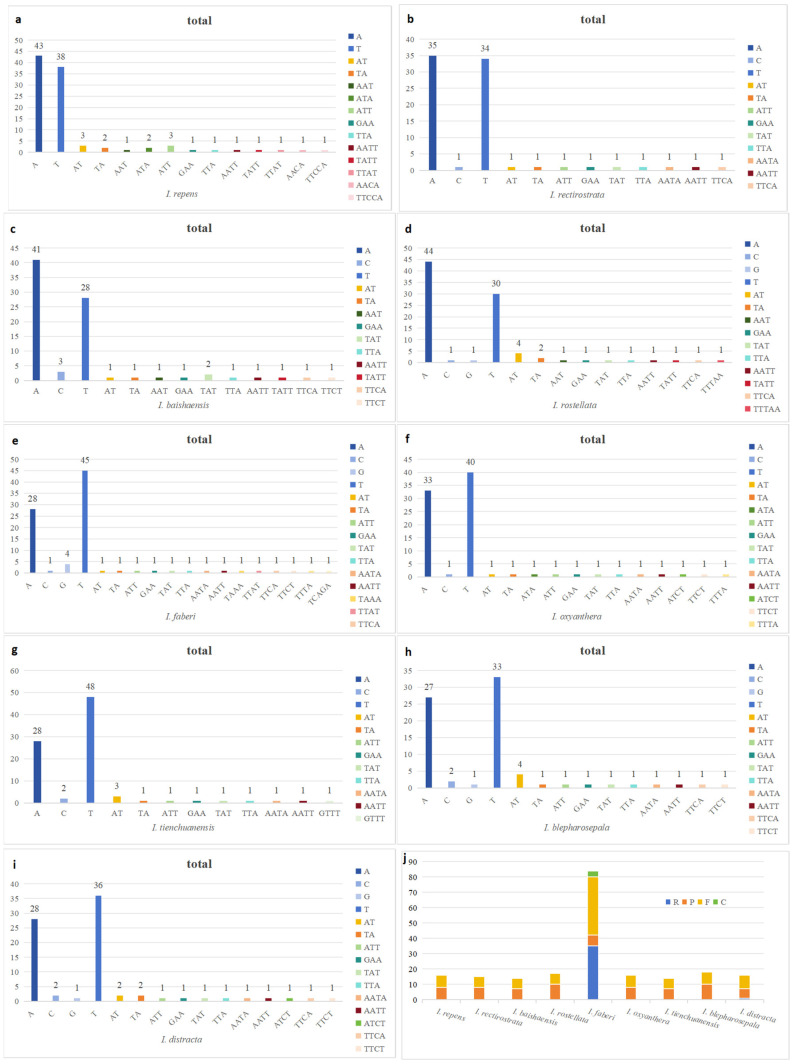
SSR locus analysis of nine *Impatiens* species chloroplast genomes. (**a**–**i**) Frequencies of identified SSR motifs in different repeat class types. (**j**): Numbers of different SSR types detected in the nine *Impatiens* species.

In this study, SSR analysis of nine species of *Impatiens* plants yielded a total of 778 SSR sequences, which were 661 mononucleotides, accounting for 85.0% of the total; 36 dinucleotides, accounting for 4.6% of the total; 41 trinucleotides, accounting for 5.3% of the total; 36 tetranucleotides, accounting for 4.6% of the total; and 4 pentanucleotides, accounting for 0.5% of the total; hexanucleotides were not detected; the highest number was of single-nucleotide sequences, followed by trinucleotide sequences, and the lowest number was of pentanucleotides; the number of SSRs in *I. repens* was the highest, 99, of all the species, and the lowest was 76 in *I. blepharosepala,* as shown in Figure 3a–i.

Scattered repeat sequences in the chloroplast genomes of nine *Impatiens* species were counted, and the results are shown in Figure 3j. A total of 210 pairs of repetitive sequences were identified, specifically: 36 pairs of reverse repetitive sequences (R), 71 pairs of palindromic repetitive sequences (P), 99 pairs of forward repetitive sequences (F), and 4 pairs of complementary repetitive sequences (C). Among these species, *I. faberi* had the highest number of repetitions, totalling 84, including 35 forward and 7 palindromic repeats, while *I. baishaensis* and *I. tienchuanensis* had the lowest number of repetitions. The highest number of palindromic repeats was found in *I. rostellata* and *I. blepharosepala*. The highest number of forward repeats was also found in *I. faberi*. Overall, palindromic repeats accounted for about 33.80% of the total number of scattered repeats, while forward repeats accounted for about 47.14%. It is noteworthy that the reverse repeat sequences were found only in *I. faberi* and *I. distracta*, while complementary repeat sequences were detected only in *I. faberi*.

### 2.4. IR Expansion and Contraction

**Figure 4:**
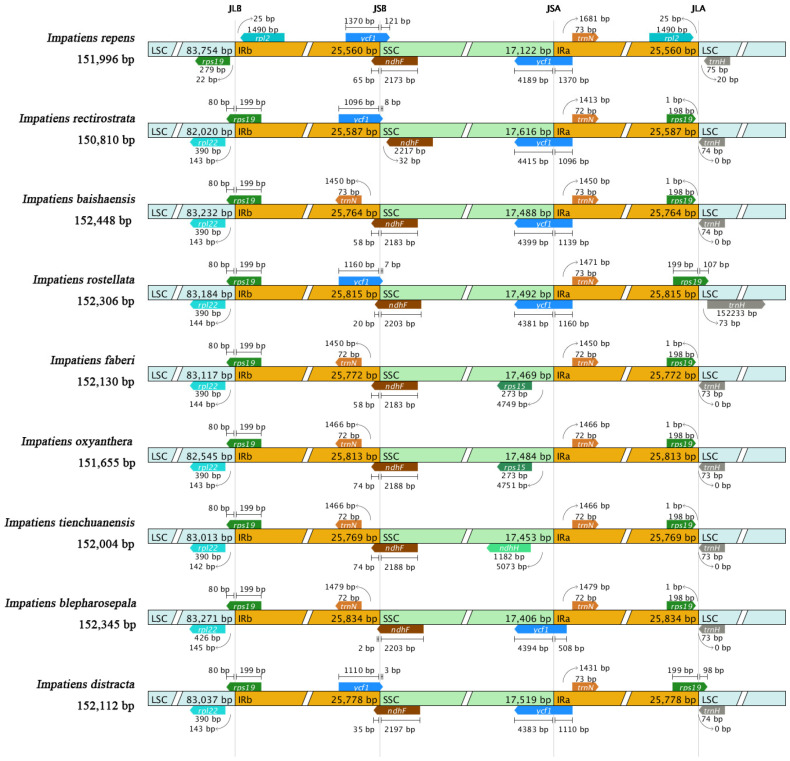
Comparisons of the borders of LSC, SSC, and IR regions among nine *Impatiens* chloroplast genomes.

An in-depth analysis of the IR boundary regions of the chloroplast genomes of nine *Impatiens* species showed that these regions exhibited a high degree of conservation in terms of gene number and sequence length and were all tetrameric in structure. Nevertheless, some variability was observed in the boundary regions. Specifically, the length of the IR regions of the nine *Impatiens* plants ranged from 25,560 bp (*I. repens*) to 25,834 bp (*I. blepharosepala*). In terms of chloroplast genome structure, the *trnH* gene is consistently located in the LSC region; two copies of the *rps19* gene are located immediately adjacent to the LSC/IRB and LSC/IRA boundaries, respectively, and it is noteworthy that one copy of the *rps19* gene is missing from the LSC/IRA boundary in *I. repens*. In addition, the *rpl2* gene is situated entirely within the IR region and closer to the LSC boundary, a feature found only in *I. repens*. *rpl22* gene, on the other hand, is situated in the LSC region and is missing in *I. repens*. *ndhF* gene is close to the SSC and IRB boundary, whereas the *trnN* gene is situated entirely in the IR region and is missing in *I. baishaensis*, *I. faberi*, *I. oxyanthera*, *I. tienchuanensis,* and *I. blepharosepala*. *ycf1* gene is present in two copies in *I. repens*, *I. rectirostrata*, *I. baishaensis*, *I. rostellata*, *I. blepharosepala,* and *I. distracta* was detected, and it usually spanned the SSC versus IR region, but gene scaling in the IR/SC border region varied among species, as shown in Figure 4. In addition, the *rps15* gene was found only in *I. faberi* and *I. oxyanthera* and was located in the SSC region, while the *ndhH* gene was present only in *I. tienchuanensis*, also located in the SSC region.

### 2.5. Analysis of Chloroplast Genome Sequence Divergence in Balsaminaceae Species

**Figure 5:**
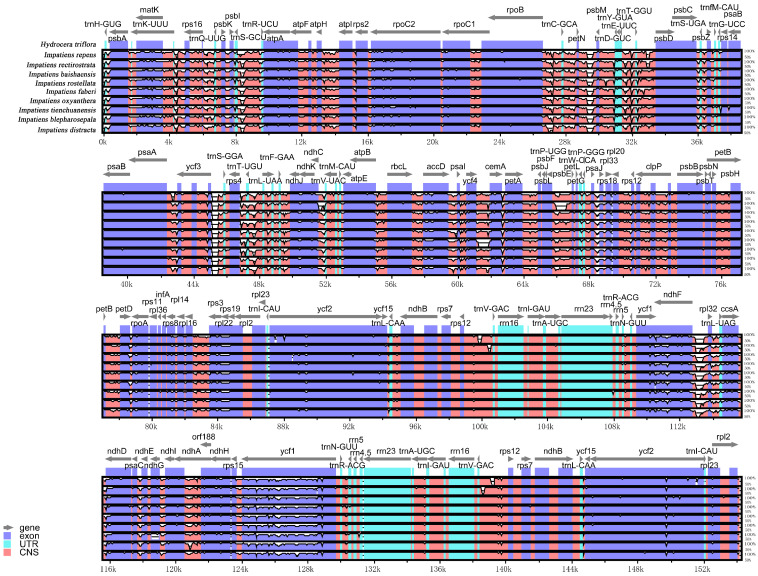
Comparative plots based on sequence identity of chloroplast genome of nine *Impatiens* species, using *Hydrocera triflora* as the reference genome.

Interspecific comparison of sequence identity among the chloroplast genomes of nine *Impatiens* species using mVISTA (https://genome.lbl.gov/vista/mvista/submit.shtml (accessed on 2 August 2024)) with *Hydrocera triflora* as a control showed that the chloroplast genome sequences of the nine *Impatiens* plants were highly similar, conserved, and homologous across species, with sequences of coding regions being more conserved among species and non-coding region sequences relatively more varied, and the IR region was also more conserved than the SC region (Figure 5). Within the coding genes as well as the spacer regions, some degree of highly differentiated regions were observed, such as *trnS-GCU*, *psbM*, *ycf3*, *trnT-UGU*, *ycf4*, *psbE*, *ndhF*, *ndhA,* and *ycf1*. Compared to *Hydrocera triflora*, the *psbM* gene showed the greatest variation within the LSC region, while the *ycf1* gene showed the most significant variation in the SSC region, and the *ndhF* gene showed a high degree of divergence in the IR region.

### 2.6. Basic Characteristics of Highly Variable Area Segments

**Figure 6:**
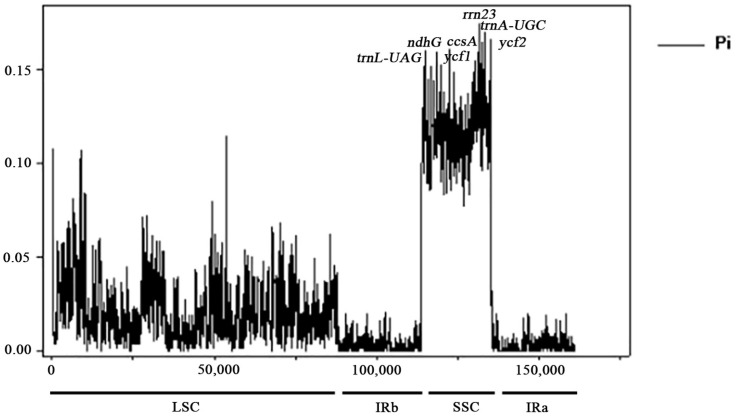
Sliding-window analysis of the whole chloroplast genomes of nine *Impatiens* species. Window length: 100 bp; step size: 25 bp. X-axis: position of the midpoint of a window. Y-axis: nucleotide diversity of each window.

Nucleotide polymorphism (Pi) is an important indicator of the degree of variation in nucleic acid sequences of different species, and highly variable regions can provide valuable molecular markers for population genetics studies. The analysis of nucleotide polymorphisms conducted in this study (Figure 6) revealed significant sequence divergence in the SSC region, in contrast to lower nucleotide polymorphisms in the LSC and IR regions. Analysis of nucleotide diversity (Pi) values of coding and intergenic regions of Balsaminaceae plants revealed that the Pi values of intergenic regions were higher than the coding regions, implying more drastic differentiation of intergenic regions. The mean nucleotide diversity (Pi) value was 0.02433 in 10 species of Balsaminaceae plants, in which seven loci, *trnL-UAG*, *ndhG*, *ycf1*, *ccsA*, *rrn23*, *trnA-UGC*, and *ycf2*, as the hotspots of mutation, exhibited abnormally high Pi values (>0.13); the *rrn23* gene had the highest Pi value, 0.17356. Combined with the results of DnaSP and mVISTA analyses, these highly differentiated loci can be used as potential molecular markers for the identification of closely related species and for phylogenetic studies.

### 2.7. Selection Pressure Analysis

**Figure 7:**
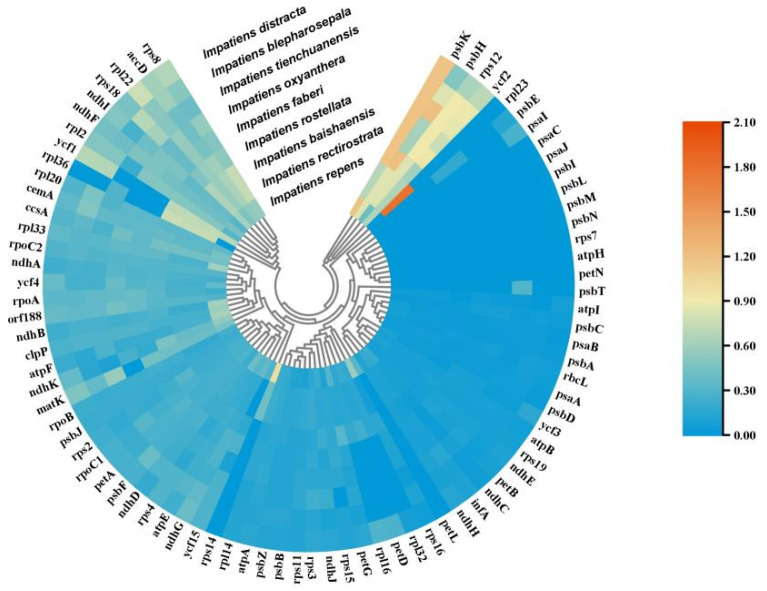
Selective pressure analysis results. Cluster heatmap showing the Ka/Ks values of chloroplast genomes from nine species, using *Hydrocera triflora* as a reference; the Ka/Ks value varies between 0 and 2.1, corresponding to a colour range of blue to red.

Selection pressure is also known as evolutionary pressure and can generally be classified as positive, neutral, and purifying selection. In genetics, Ka/Ks or dN/dS represents the ratio between heterozygous substitutions (Ka) and homozygous substitutions (Ks). Ka = number of SNPs in which non-synonymous substitutions occur/number of non-synonymous substitution loci, and Ks = number of SNPs in which homozygous substitutions occur/number of homozygous substitution loci. By analysing the Ka/Ks ratio, it is possible to assess whether protein-coding genes are affected by the selection pressure. Specifically, when the Ka/Ks ratio is greater than 1, it indicates a positive selection effect; when it is equal to 1, it indicates neutral selection; and when it is less than 1, it indicates purifying selection. In this study, we calculated the Ka/Ks values of 81 protein-coding genes in nine *Impatiens* species using *Hydrocera triflora* as a benchmark (Figure 7). The results showed that only a few genes were under positive selection in *Impatiens* species, while the vast majority of genes were under purified selection, and no neutrally selected genes were detected. The *petL*, *psaI,* and *psbT* genes were only detected individually in *I. repens*, *I. distracta,* and *I. tienchuanensis,* respectively. *psbH* genes had Ka/Ks values > 1 in *I. baishaensis*, *I. rostellata,* and *I. tienchuanensis* species, and *psbK* genes had Ka/Ks values >1 in *I. repens*, *I. rostellata*, *I. faberi*, *I. oxyanthera*, *I. tienchuanensis*, *I. blepharosepala,* and *I. distracta* species; Ka/Ks values for the *rpl23* gene were >1 in *I. rectirostrata*, and Ka/Ks value of *rps14* gene were >1 in *I. repens* species, indicating that they are positively selected.

### 2.8. Phylogenetic Analysis of Balsaminaceae

**Figure 8:**
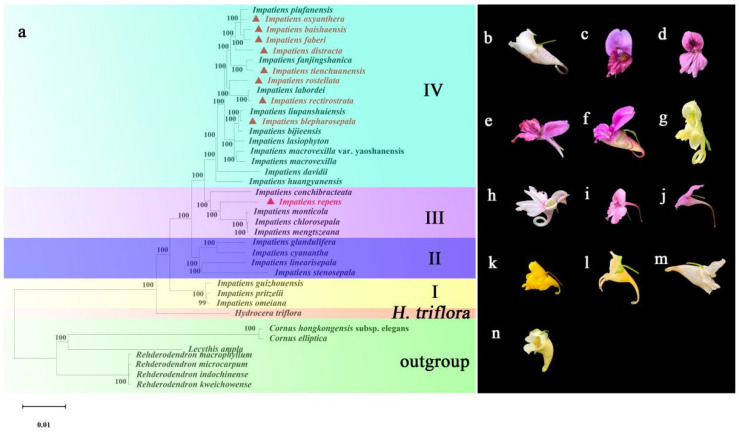
Phylogenetic tree based on chloroplast genome sequences of thirty-one Bromeliaceae species and seven other related species using the NJ method. (**a**) Text in bold red preceded by red triangles indicates the nine *Impatiens* species studied here: (**b**) *I. oxyanthera*; (**c**) *I. faberi*; (**d**) *I. baishaensis*; (**e**) *I. distracta*; (**f**) *I. tienchuanensis*; (**g**) *I. rostellata*; (**h**) *I. rectirostrata*; (**i**) *I. blepharosepala*; (**j**) *I. macrovexilla* var. yaoshanensis; (**k**) *I. repens*; (**l**) *I. chlorosepala*; (**m**) *I. guizhouensis*; (**n**) *I. pritzelii*.

Phylogenetic trees were constructed using the NJ and ML methods based on the chloroplast genome sequences of 38 plant species (Figure 8 and Figure S9), and it was found that the phylogenetic trees constructed with the two methods maintained a high degree of consistency, dividing all the species of *Impatiens* into four branches (I to IV), with slightly different bootstrap (BS) values for each tree topology. *I. guizhouensis*, *I. pritzelii*, and *I. omeiana* were clustered into branch I; *I. glandulifera*, *I. cyanantha*, *I. linearisepala*, and *I. stenosepala* were clustered into branch II; and *I. repens*, *I. monticola*, *I. chlorosepala* and *I. mengtszeana* were clustered into branch III, *I. rectirostrata*, *I. baishaensis*, *I. rostellata*, *I. faberi*, *I. oxyanthera*, *I. tienchuanensis*, *I. blepharosepala*, *I. distracta*, etc., were clustered into branch IV. In terms of leaf blade type, all *Impatiens* plants have alternate leaves; lateral sepals are either four or two, with four lateral sepals in all species of basal I, followed by a gradual evolution to two sepals in II-IV; the flag petals are rounded, obovate, subreniform, elliptic, and broadly ovate; the pterostigma is of three types, stipitate, sessile, and subsessile, with a mixture of flagellar shape and the presence or absence of stipes on the pterostigma in each subgroup. The labellum is funnel-shaped, saccate, cupular, boat-shaped, and angular, and its classification among the branches is not obvious. Basal taxon I is basically fusiform, and the ovaries in II to IV are fusiform or linear. Capsules are rod-shaped, fusiform, linear, barred, and oblong, with the basal taxon I basically rod-shaped and all five shapes distributed in taxons II to IV. In the phylogenetic tree, all nodes had BS values of 99% or 100%.

## 3. Discussion

### 3.1. Chloroplast Genome Structure

In this study, nine *Impatiens* species plant chloroplast genomes were obtained using Illumina MiSeq (Illumina Corporation, San Diego, CA, USA), which provides an important resource for genetic engineering as well as evolutionary and species identification studies. The obtained *Impatiens* chloroplast genomes all exhibited a typical angiosperm tetragonal structure, which usually consists of an LSC, an SSC, and two IR regions [30,31]. The size of the chloroplast genomes of the nine *Impatiens* species ranged from 150,810 bp to 152,345 bp, which demonstrates the highly conserved nature of the *Impatiens* chloroplast genomes. Although the chloroplast genomes of angiosperms evolve relatively rapidly and may be accompanied by inversions and gene loss [32], the coding genes, tRNAs, and rRNAs of the nine *Impatiens* species remain largely identical in terms of gene composition, with only minor differences. Overall, these nine chloroplast genomes contained 130 (*I. oxyanthera* and *I. tienchuanensis*) to 166 (*I. distracta*) genes, with 85 protein-coding genes (*I. oxyanthera* and *I. tienchuanensis*) to 105 (*I. distracta*); 37 (*I. baishaensis* et al.) to 50 (*I. repens*) tRNA genes; and 8 (*I. baishaensis* et al.) to 12 (*I. repens* et al.) rRNA genes. The present study revealed that two additional copies of the *rpl2* gene were present in the chloroplast genome of *I. repens*, while the *rpl22* gene was deleted, which marked an insertion and deletion event of the gene during the evolutionary history of *I. repens*. In addition, the chloroplast genomes of *I. repens*, *I. rectirostrata*, *I. baishaensis*, *I. rostellata*, and *I. distracta* were all missing a copy of the *trnN* gene. Previous studies have also indicated that deletions of the *lhbA*, *infA*, *rpl22,* and *rps16* genes, as well as introns and copies of the *rpl2*, *clpP,* and *rps12* genes, have been reported in the chloroplast genomes of other plants [33,34].

### 3.2. Codon Preference, SSR, and Long Repeat Structure Analysis

Codon preference refers to the tendency to select different codons in genetic coding, which is critical for protein synthesis and thus influences gene regulation and evolution at the molecular level [35]. The nine *Impatiens* plants had a total of 32–36 codons with relative synonymous codon usage (RSCU ≥ 1), most of which ended in A/U and very few in C/G. This result is similar to that of *Hellenia speciosa* [36], *Actinidia latifolia* [37], and *Vitis heyneana* [38], among others, which prefer codons ending in A/U. Codon usage was relatively similar in the chloroplast genomes of the nine *Impatiens* species, suggesting that these nine *Impatiens* species may have experienced similar environmental stresses during their evolution. The study of codon preference may lay a theoretical foundation for subsequent gene expression and molecular evolution studies in *Impatiens* plants.

SSRs are abundant, highly polymorphic, uniformly cover the entire genome, are co-dominant and simple to detect, and thus are widely used as second-generation molecular markers in the fields of genetic map construction, target gene localisation, genetic diversity studies, molecular-assisted breeding, and germplasm resource identification [39,40]. In this study, four to five nucleotides were found in the chloroplast genomes of nine *Impatiens* plants, and these SSRs were mainly distributed in the LSC region, while the fewest SSRs were found in the IRs region, and the single nucleotide was the most common among all SSRs, which is in line with the findings of other scholars [41,42]. Long repetitive sequences are believed to play an important role in genome recombination and rearrangement and also contain phylogenetic information in some populations [43]. Also, due to abnormal slip-strand mismatches and recombination, repetitive sequences have an important role in genomic variation and rearrangement processes [44]. In the present study, repeat analysis of nine *Impatiens* plant genomes detected 210 repetitive sequences, most of which were 30–39 bp in length. The highest number of repetitive sequences was found in *I. faberi* among the nine *Impatiens* plants. The number, type, and length of scattered repeat sequences vary from species to species, and these differences can provide a theoretical basis for the development of molecular genetic markers in plants [45].

### 3.3. IR Expansion and Contraction

The IR region of the chloroplast genome is usually regarded as the most conserved part; however, the sequence of the IR and SC boundary region may undergo outward expansion or inward contraction, which can lead to the increase or decrease in the copy number of the relevant genes or the formation of pseudogenes in the boundary region, which is a common phenomenon in the evolution of the chloroplast genome and is the main factor contributing to the difference in its length [46]. For example, the *Tetracentron* chloroplast genome shows expansion/contraction events in the IR region [47], while the genome of *Veroniceae* shows a duplication of the *rps19* gene in the IR region [48]. Expansion of the IR region has been found in the *Vigna mungo* chloroplast genome [49], whereas a gene loss event was found in *Sorghum sudanense* chloroplast genome [50]. Yang [51] showed that eight species in the same family as *Persicaria lapathifolia* had *trnH* and *psbA* located in the LSC region, which to some extent validates the conservation of the angiosperm chloroplast genome. The *trnH* genes at the LSC/IRa boundary underwent significant boundary expansion and contraction, and all *trnH* genes were located in the LSC region, with a deletion of a gene related to the ribosomal protein *rps19*, which could enhance photosynthetic efficiency and reduce protein synthesis efficiency to a certain extent [52]. Comparison of the chloroplast genomes of nine *Impatiens* species revealed that the boundary region between the LSC and IRb regions is relatively conserved, and the distribution and specific locations of genotypes in the LSC region are similar. Compared to the other eight *Impatiens* species, the IR region of *I. repens* showed contraction with the smallest length (25,560 bp), mainly because the *rps19* gene located at the LSC/IR boundary enlarged the LSC region by 279 bp. The absence of the *rpl22* gene in the LSC region was inconsistent with the other eight *Impatiens* species. In addition, the gene *ndhH* for the NADH dehydrogenase subunit was found only in the SSC region of *I. tienchuanensis*. Studies have also been conducted to observe the loss of the *ndh* gene in Orchidaceae [53], *Genlisea* [54], and *Selaginella* [55], suggesting that it may be dispensable for some photosynthetic autotrophic plant species, and since most of the samples of the nine species sequenced in this paper were taken from the field and belong to different populations, the variation in the loss of the *ndhH* gene is understandable. In addition, the GC content of the nine chloroplast genomes showed an uneven distribution, as evidenced by the fact that the GC content of all IR regions was higher than that of the LSC and SSC regions, which was most likely due to the relatively high GC content of the four rRNAs in the IRs region [56,57]. Contraction and expansion of IR region boundaries are common evolutionary events in chloroplast genomes and are the main cause of changes in chloroplast genome length, which plays an important role in evolution [58].

### 3.4. Highly Variable Areas and Selection Pressure Analysis

Plant taxonomic evolution and genetic development are based on molecular markers, such as highly mutated regions, SSRs, and SNPs. *ndhA* and *ndhH* have been widely used in taxonomic and molecular phylogenetic studies as genes encoding NADH dehydrogenase subunits [59]. In this study, seven highly differentiated gene fragments, including *trnL-UAG*, *ndhG*, *ycf1*, *ccsA*, *rrn23*, *trnA-UGC*, and *ycf2*, were identified through the excavation of highly mutated regions of the intact chloroplast genomes of nine Balsaminaceae plants, among which *ycf1* has been widely used in phylogenetic analyses among wild *Impatiens* species [60], and it has been shown that *rpl32-trnN* has likewise been used in phylogenetic studies of *Impatiens* plants [42], whereas the results of this paper found that the degree of divergence in this region was not prominent and the degree of differentiation was not high. This may be due to the complex evolutionary issues in *Impatiens* plants, which are particularly difficult to classify and identify. Therefore, the highly mutated region screened in this paper to serve as a potential molecular marker for *Impatiens* plants can help to provide rich discriminatory information for the identification of new species of *Impatiens* plants as well as to elucidate phylogenetic, molecular evolutionary, genetic developmental, and phylogenetic relationships among species.

According to the results of sequence differentiation analysis, the variation in LSC and SSC regions was significantly higher than that in IR region, which was presumed to be related to the selection pressure, which was less likely to lead to structural variation and more likely to be relatively stable. In the nine *Impatiens* species, the Ka/Ks values of the genes encoding *psbH*, *psbK*, *rpl23,* and *rps14* were greater than 1 in response to the evolutionary selection of the genes, suggesting that positive selection has occurred for the above genes. *psbH* and *psbK* are integral parts of the PSII complex, and *psbK* is likely to be involved in the assembly and stability of the PSII complex [61]. The adaptive evolution of two genes related to photosynthesis in this study suggests that these two genes may be involved in the adaptation of *Impatiens* species to different light habitats. *rpl23* and *rps14* both belong to the ribosomal protein gene family, and it has been shown that high expression of *rpl23a* leads to significant increases in the fresh weight, root length, proline, and chlorophyll content of rice seedlings [62]. *rps14* is involved in nucleic acid metabolic processes and affects RNA post-transcriptional processing, which in turn inhibits the growth of Arabidopsis stem cells, causing plants to exhibit dwarf characteristics [63]. Therefore, it is hypothesised that they are positively selected to resist abiotic stress tolerance traits and thus adapt to the different environments in which the plants are found. Taken together, these positively selected genes may have played a key role in the adaptation of *Impatiens* plants to various environmental conditions, especially to different light habitats. The adaptive evolutionary analysis of the chloroplast genome of *Impatiens* plants has deepened the in-depth understanding of genes containing positive selection loci, provided a basis for the discovery of functionally important chloroplast genes, and provided certain implications for the future innovation of germplasm resources for species in this genus.

### 3.5. Phylogenetic Analysis

Due to the different rates of molecular evolution in different regions, the relationships among the species of Balsaminaceae are complex, controversial, and difficult to classify. In this study, thirty-eight species were phylogenetically analysed, including thirty-six species of Rhododendron and two species of Cornus, among which, all thirty species of Balsaminaceae were clustered together, and *H. triflora* was at the basal-most position of the branch, which was in a sister genus relationship with *Impatiens*, further supporting that Balsaminaceae is a monophyletic group, which is in agreement with Zhao [64]. *I. guizhouensis*, *I. pritzeli*, and *I. omeiana* were at the base of the *Impatiens* phylogenetic tree in this study, which may be due to the fact that their chloroplast genomes retained the original species status of *Impatiens*, which is similar to the results of Luo [42]. In the previous taxonomy by Yu [1], the Chinese *Impatiens* plants were explored in depth taxonomically under the genus based on the number of carpels, fruit species, inflorescence characters, and flower morphology, together with the microstructure of leaf epidermis, pollen morphology, microfacets of seed coat, and molecular biological evidence (including ITS and *atpB-rbcL* sequences), and were finally classified into 8 groups. In this paper, *I. repens* belongs to sect. *Fusicarpa* of *Impatiens*, clustered with *I. monticola*, *I. chlorosepala*, and *I. mengtszeana*, which are characterised by alternate or opposite leaves, small petioles borne in the axils of the upper leaves, and short fusiform fruits, most of which are peritrichous, with numerous seeds. The taxonomic status of *I. repens* has not been clearly defined in the current study, and more in-depth studies are needed to expand the sample size. At the same time, phylogenetic studies on *Impatiens* need to be strengthened in order to construct a taxonomic system that can be widely applied to *Impatiens* species around the world. The other eight *Impatiens* species studied here are clustered in the same branch IV, belonging to sect. *Laxiflora*, and they are characterised by a 5-carpellate ovary with a long common pedicel, two lateral sepals, the apical part of the distal lobes of the winged petals not filamentous, and a narrowly linear capsule. Among them, *I. baishaensis* is a new species discovered in 2017, and from the phylogenetic tree, it seems to be clustered with *I. piufanensis*, *I. oxyanthera*, and *I. faberi*, which is the same as Ding’s findings that *I. baishaensis* and *I. oxyanthera* have similar morphological characters similarly [65], but the study in this paper shows closer affinity with *I. faberi*, and the chloroplast genome can provide rich genetic information with high resolution, which helps to define the affinity and evolutionary history between species more precisely; so, this paper further confirms the taxonomic position of *I. baishaensis* in *Impatiens*. In addition, there is a close relationship between flower colour, flower spot, flower type, and reproduction [66]. These characters evolved gradually during plant evolution to adapt to different ecological environments and reproductive strategies. For plants, these features not only help to attract pollinators and improve reproductive success but may also play an important role in protecting flowers from predators and adapting to specific environments [67]. The flowers of *Impatiens* plants usually appear yellow and pinkish purple, which will help pollinators such as bees and moths to pollinate and reproduce them, and the flower spots of *Impatiens* plants may also be for attracting pollinators [68]; we all know that in most of the *Impatiens* plants exists the structure of flower spacing, and the nectar stored in the spacing is more attractive to the pollinators, and the length of the flower spacing may be the result of co-evolution with pollinators [69]. From the phylogenetic tree in Figure 8, it is clear that the sepals of *Impatiens* species have evolved from the original four lateral sepals to two sepals, although the leaves, flag petals, wing petals, labellums, and seeds are not clearly divided into branches according to their shapes, which precisely indicates that the taxonomy needs, firstly, the collection of larger-scale molecular data, which will lead to the resolution of a more complete phylogenetic tree; and, secondly, the use of high-throughput sequencing technology to reveal the relationship between microbial diversity and ecosystems, which will lead to the development of a better phylogenetic tree. Second, the use of high-throughput sequencing technology to reveal microbial diversity and ecosystem functions; third, an in-depth understanding of species evolution to provide a scientific basis for biodiversity conservation; and the construction of a standardised and globalised database of taxonomic information to promote data sharing and cooperation.

## 4. Materials and Methods

### 4.1. Plant Material, DNA Extraction, and Sequencing

Nine *Impatiens* plant samples were collected for this paper. Details of the samples are given in Table S18, where not only waxy leaf specimens but also fresh leaves were collected in the field, washed, and packed in self-sealing bags with colour-changing silica gel desiccant for DNA extraction and subsequent studies. Total genomic DNA was extracted using the mCTAB method [70], integrity was examined by 1.2% agarose gel electrophoresis, and DNA concentration and quality were assessed by a nucleic acid protein detector. Sequencing was performed using the company’s Illumina NovaSeq (Illumina Corporation, San Diego, CA, USA) 6000 device for double-end sequencing of the chloroplast genomes of nine *Impatiens* species. Sequencing clean data was attained by removing the pairwise readings with adapter and single-ended sequencing reads with N content exceeding 1/10 of the full length and the pairwise readings with low-quality base numbers (Q ≤ 5) in single-ended sequencing reads exceeding 1/2 of the full length.

### 4.2. Chloroplast Genome Assembly and Annotation

The chloroplast genomes of the nine *Impatiens* species were assembled using GetOrganelle 1.7.5.0 [71], the default parameters were selected to obtain the complete cyclic chloroplast genome sequences, the fasta format files obtained from the assemblies were submitted to the online annotation website Cpgavas2 (http://47.96.249.172:16019/analyzer/home (accessed on 18 July 2024)) [72], and then the chloroplast genome-related sequence information was obtained.

The GC contents of the four regions of the chloroplast genomes of the nine *Impatiens* plants were counted using an online tool (http://cloud.genepioneer.com:9929 (accessed on 21 July 2024)) [73]. And the chloroplast genome atlas was generated by uploading the annotated gbf files using the online Chloroplot (https://irscope.shinyapps.io/Chloroplot/ (accessed on 23 July 2024)) software [74].

### 4.3. Codon Preference Analysis

The relative synonymous codon usage of the included protein-coding gene sequences was statistically analysed using Codon W 1.4.2 [75] and visualised via TBtools-II software.

### 4.4. Repeat Sequence Analysis

The chloroplast genomes of nine *Impatiens* species were analysed for simple repeat sequences using the online software MISA (https://webblast.ipk-gatersleben.de/misa/index.php (accessed on 26 July 2024)) [76], with the order of the one to six nucleotide repeat unit parameters set at 10, 6, 4, 3, 3, 3, 3, and the minimum distance value between the 2 SSRs was set to 100 bp. An online software called REPuter (https://bibiserv.cebitec.uni-bielefeld.de/reputer (accessed on 28 July 2024)) [77] was used to count the cpDNA scattered repeat sequences of the nine *Impatiens* plant species. In the repeated sequences, the specific parameters were set as follows: the value of minimum repeated sequence was entered as 30 bp, the value of Hamming distance was entered as 3, and the value of sequence identity was entered as 90%.

### 4.5. Comparative Analysis of IR Regions

Variability in the location of the region was analysed by comparing the border region of the large single copy region, the reverse repeat, and the small single copy region of the cpDNAs of nine *Impatiens* plant species by the online tool IRscope (https://irscope.shinyapps.io/irapp/ (accessed on 29 July 2024)) [78].

### 4.6. Genome Sequence Divergence Between Chloroplast Genome Species

Using an online tool, mVISTA (https://genome.lbl.gov/vista/mvista/submit.shtml (accessed on 2 August 2024)) [79], the cpDNA of *Hydrocera triflora* was used as a reference sequence to visualise and analyse nine *Impatiens* plants, which reflected conserved versus variant regions among species by comparing the differences between exons, introns, non-coding, and coding regions of the chloroplast genome.

### 4.7. High-Variance Regional Analyses

Chloroplast genome highly variable regions were analysed using MAFFT-7.526 software [80], using cpDNA of *Hydrocera triflora* as a reference sequence for comparison, followed by MEGA 11 [81] for manual correction and DnaSP 5 software for nucleotide diversity analysis [82] with a step size of 25 bp and a window length of 100 bp to explore highly variable regions in the chloroplast genomes of the nine *Impatiens* species.

### 4.8. Selection Pressure Analysis

BLASTN (2.10.1) was used to compare other protein sequences with the reference protein sequence to determine the best matches and thus obtain the homologous protein sequence. Subsequently, MAFFT-7.526 software was used to achieve automatic alignment of homologous protein sequences. The aligned protein sequences were mapped to the corresponding coding sequences through Perl scripting technology to generate the aligned coding sequences. Next, the KaKs_Calculator2 tool (http://112.86.217.82:9929/#/tool/alltool/detail/305 (accessed on 5 August 2024)) [83] was used to calculate ka and ks values. Finally, Excel was used to perform the data statistics, and the visual presentation of the data was achieved with the help of TBtools.

### 4.9. Phylogenetic Analysis

Chloroplast genome sequences of 36 species of Rhododendron and 2 species of Cornaceae plants, of which 4 Styracaceae, 1 Lecythidaceae, and 2 Cornaceae plants were used as outgroups, were used for chloroplast genome sequence comparison using MAFFT-7.526 software. Then, a phylogenetic tree was constructed based on NJ and ML methods in MEGA 11 software [81], where the self-expansion value of each parameter was set to 1000.

## 5. Conclusions

The study of chloroplast genomes lays an important foundation for plant species identification, origin tracing, evolutionary history exploration, genetic diversity analysis, and resource conservation and utilisation. In this study, we completed the sequencing and assembly of the complete chloroplast genomes of nine *Impatiens* species. Comparative genome analysis revealed that the chloroplast genomes of *Impatiens* are relatively conserved in structure, displaying typical tetragonal ring-like features. The variations in genome length mainly originated from differences in the IR/SSC and LSC/IR boundary regions. In addition, the chloroplast genomes of these nine *Impatiens* species tended to end in A/U in terms of relative synonymous codon usage frequency (RSCU), which coincided with their lower GC-content genomic features. In this paper, 778 SSR loci were identified that can be used as molecular markers for future intraspecific diversity studies in *Impatiens* plants. Phylogenetic relationships within, between groups, and between species of *Impatiens* were determined by constructing a phylogenetic tree using NJ and ML methods. Balsaminaceae is a monophyletic taxon, and *H. triflora* is at the base of the branch and is the original species of Balsaminaceae, which is in a sister-group relationship with *Impatiens* species. In this paper, a comparative analysis of intact chloroplasts from nine species of *Impatiens* plants provides valuable new insights for exploring the structure and evolutionary pathways of plant bodies. Accordingly, the application of intact chloroplast genomics can facilitate species identification, precise definition of taxonomy, and in-depth analyses of genome evolutionary history. In order to explore the affinities within the Balsaminaceae in greater depth, morphological observations should be combined with genome-wide analyses to improve our understanding of evolutionary history.

## Figures and Tables

**Table 1:** Summaries of complete chloroplast genomes of nine *Impatiens* species.

Latin Name	Total Length (bp) and GC (%)		LSC Region Length (bp) and GC(%)		SSC Region Length (bp) and GC(%)		IR Region Length (bp) and GC (%)		Total Genes	Total CDS	Total rRNA	Total tRNA
*I. repens*	151,996	37	83,754	34	17,122	29	25,560	43	147	94	12	41
*I. rectirostrata*	150,810	37	82,020	34	17,616	29	25,587	43	165	104	12	49
*I. baishaensis*	152,248	37	83,232	34	17,488	29	25,764	43	132	87	8	37
*I. rostellata*	152,306	37	83,184	34	17,492	29	25,815	43	147	94	12	41
*I. faberi*	152,130	37	83,117	34	17,469	29	25,772	43	133	88	8	37
*I. oxyanthera*	151,655	37	82,545	34	17,484	29	25,813	43	130	85	8	37
*I. tienchuanensis*	152,004	37	83,013	34	17,453	29	25,769	43	130	85	8	37
*I. blepharosepala*	152,345	37	83,271	34	17,406	29	25,834	43	132	87	8	37
*I. distracta*	152,112	37	83,037	34	17,519	29	25,778	43	166	105	12	49

**Table 2:** Genes in the *I. repens* chloroplast genomes.

Category	Gene Group	Gene Name
Photosynthesis	Subunits of photosystem I	*psaA*, *psaB*, *psaC*, *psaI*, *psaJ*
	Subunits of photosystem II	*psbA*, *psbB*, *psbC*, *psbD*, *psbE*, *psbF*, *psbH*, *psbI*, *psbJ*, *psbK*, *psbL*, *psbM*, *psbT*, *psbZ*
	Subunits of NADH dehydrogenase	ndhA*(2), ndhB*(2), *ndhC*, *ndhD*, *ndhE*, *ndhF*, *ndhG*, *ndhH*, *ndhI*, *ndhJ*, *ndhK*
	Subunits of cytochrome b/f complex	*petA*, petB*, petD*, *petG*, *petL*, *petN*
	Subunits of ATP synthase	*atpA*, *atpB*, *atpE*, atpF*(2), *atpH*, *atpI*
	Large subunit of rubisco	*rbcL*
	Subunits photochlorophyllide reductase	*-*
Self-replication	Proteins of large ribosomal subunit	*rpl14*, rpl16*, rpl2*(4), *rpl20*, *rpl22*, *rpl23*(2), *rpl32*, *rpl33*, *rpl36*
	Proteins of small ribosomal subunit	*rps11*, rps12**(2), *rps14*, *rps15*, rps16*, *rps18*, *rps19*, *rps2*, *rps3*, *rps4*, *rps7*(2), *rps8*
	Subunits of RNA polymerase	*rpoA*, *rpoB*, rpoC1*(2), *rpoC2*
	Ribosomal RNAs	*rrn16*(4), *rrn23*(4), *rrn4.5*(2), *rrn5*(2)
	Transfer RNAs	trnA-UGC*(2), *trnC-GCA*, *trnD-GUC*, *trnE-UUC*, *trnF-GAA*, *trnG-GCC*, trnG-UCC*, *trnH-GUG*, *trnI-CAU*(2), trnI-GAU*(4), trnK-UUU*, *trnL-CAA*(2), trnL-UAA*, *trnL-UAG*, *trnM-CAU*, *trnN-GUU*(4), *trnP-UGG*, *trnQ-UUG*, *trnR-ACG*(2), *trnR-UCU*, *trnS-GCU*, *trnS-GGA*, *trnS-UGA*, *trnT-GGU*, *trnT-UGU*, *trnV-GAC*(2), trnV-UAC*, *trnW-CCA*, *trnY-GUA*, *trnfM-CAU*
Other genes	Maturase	*matK*
	Protease	clpP1**
	Envelope membrane protein	*cemA*
	Acetyl-CoA carboxylase	*accD*
	c-type cytochrome synthesis gene	*ccsA*
	Translation initiation factor	*infA*
	other	pafI**(2), *pafII*, *pbf1*
Genes of unknown function	Conserved hypothetical chloroplast ORF	*ycf1*(2), *ycf15*(2), *ycf2*(2)

Notes: Gene*: Gene with one intron; Gene**: Gene with two introns; Gene(2): Number of copies of multi-copy genes; Gene(4): Number of copies of multi-copy genes.

## Data Availability

All raw data from this study can be downloaded from NCBI, with the corresponding accession numbers listed in the Supplementary Materials (Table S19).

## Supplementary Materials

The supporting information can be downloaded at https://www.mdpi.com/article/10.3390/ijms26020536/s1.
