# Correction: Ma et al. Complete Chloroplast Genomes of 9 *Impatiens* Species: Genome Structure, Comparative Analysis, and Phylogenetic Relationships. *Int. J. Mol. Sci.* 2025, *26*, 536

**DOI:** 10.3390/ijms26073240

**Published:** 2025-03-31

**Authors:** Hui Ma, Zhiqiang Liu, Wenxiang Lan, Mengqing Yang, Qing Mo, Xi Huang, Peiqing Wu, Haiquan Huang, Meijuan Huang

**Affiliations:** College of Landscape Architecture and Horticulture Sciences, Southwest Research Center for Engineering Technology of Landscape Architecture (State Forestry and Grassland Administration), Yunnan Engineering Research Center for Functional Flower Resources and Industrialization, Research and Development Center of Landscape Plants and Horticulture Flowers, Southwest Forestry University, Kunming 650224, China; mahui04179@swfu.edu.cn (H.M.); lzq11147890@swfu.edu.cn (Z.L.); lanwenxiang@swfu.edu.cn (W.L.); yangmq@swfu.edu.cn (M.Y.); moqing@swfu.edu.cn (Q.M.); xihuang@swfu.edu.cn (X.H.); wupeiqing@swfu.edu.cn (P.W.)

In the original publication [1], there was a mistake in the legend for Selective pressure analysis results (Figure 7). Figure 7 is supposed to be for Selection Pressure Analysis, but in the original article, Figure 7 shows Codon Preference Analysis. The correct Figure 7 appears below. The authors state that the scientific conclusions are unaffected. This correction was approved by the Academic Editor. The original publication has also been updated.

## Figures and Tables

**Figure 7:**
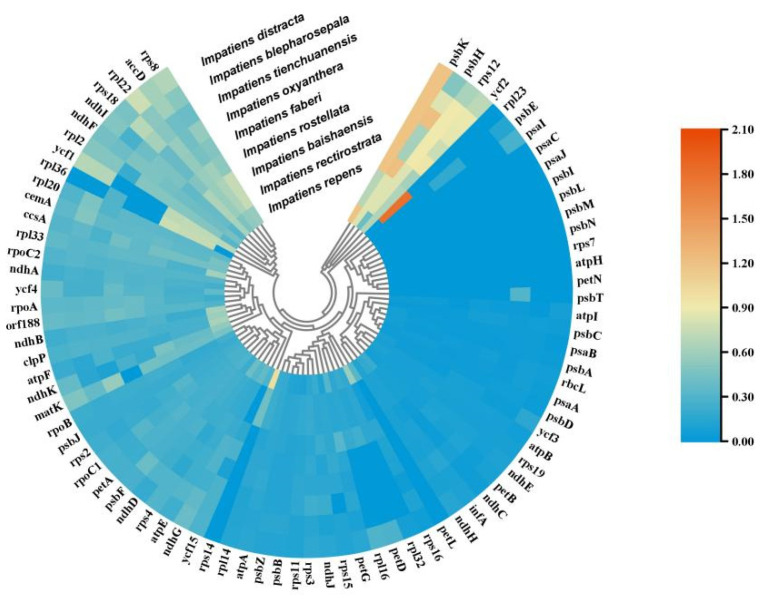
Selective pressure analysis results. Cluster heatmap showing the Ka/Ks values of chloroplast genomes from nine species, using *Hydrocera triflora* as a reference; the Ka/Ks value varies between 0 and 2.1, corresponding to a colour range of blue to red.
