# Supplementary material for: Complete Chloroplast Genomes of 9 Impatiens Species: Genome Structure, Comparative Analysis, and Phylogenetic Relationships
Source: Int J Mol Sci. 2025 Jan 10;26(2):536. doi: 10.3390/ijms26020536 (PMC11765335; doi:10.3390/ijms26020536)
Supplement: Supplementary file 1 [file ijms-26-00536-s001.zip › ijms-3369342-supplementary.pdf]

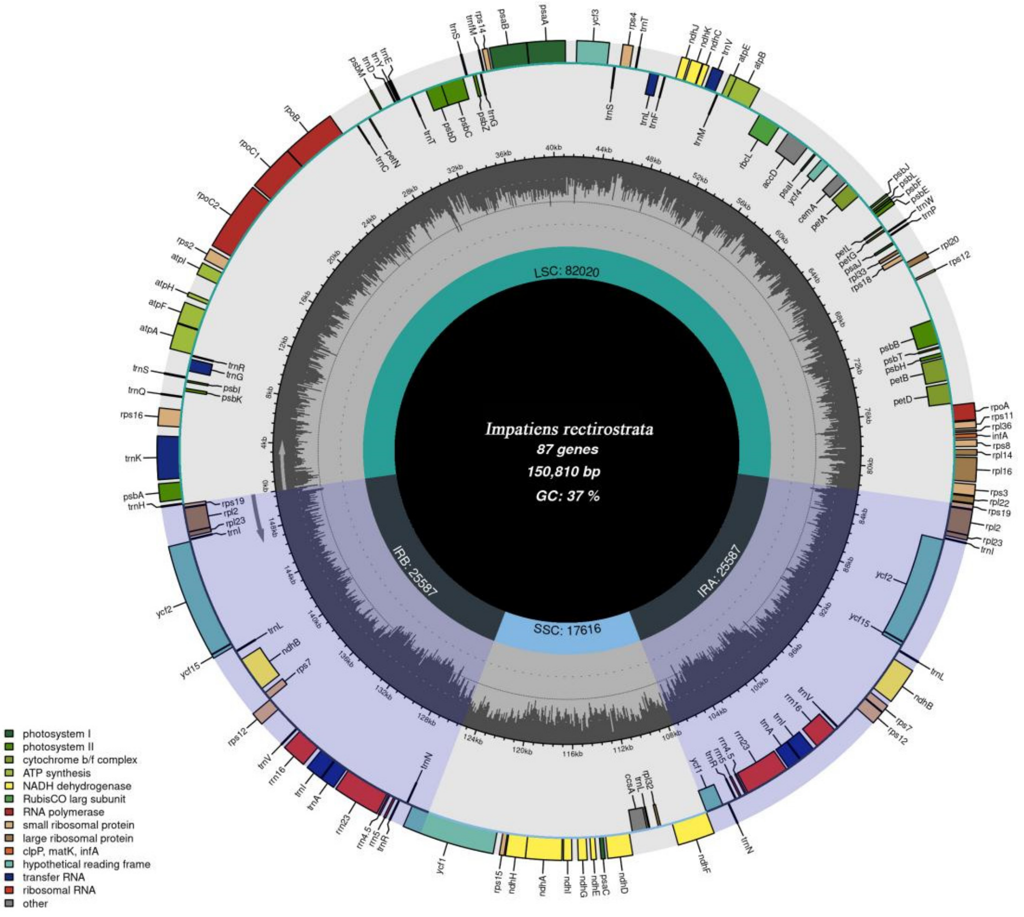

Fig. S1. Gene map of the *I. rectirostrata* chloroplast genome. Genes transcribed clockwise are shown on the outside of the outer circle, while genes transcribed counterclockwise are located inside the inner circle. Genes with different functional groups are distinguished by colour coding.The positions of the long single-copy (LSC), short single-copy (SSC), and inverted repeat regions are shown in the inner circles

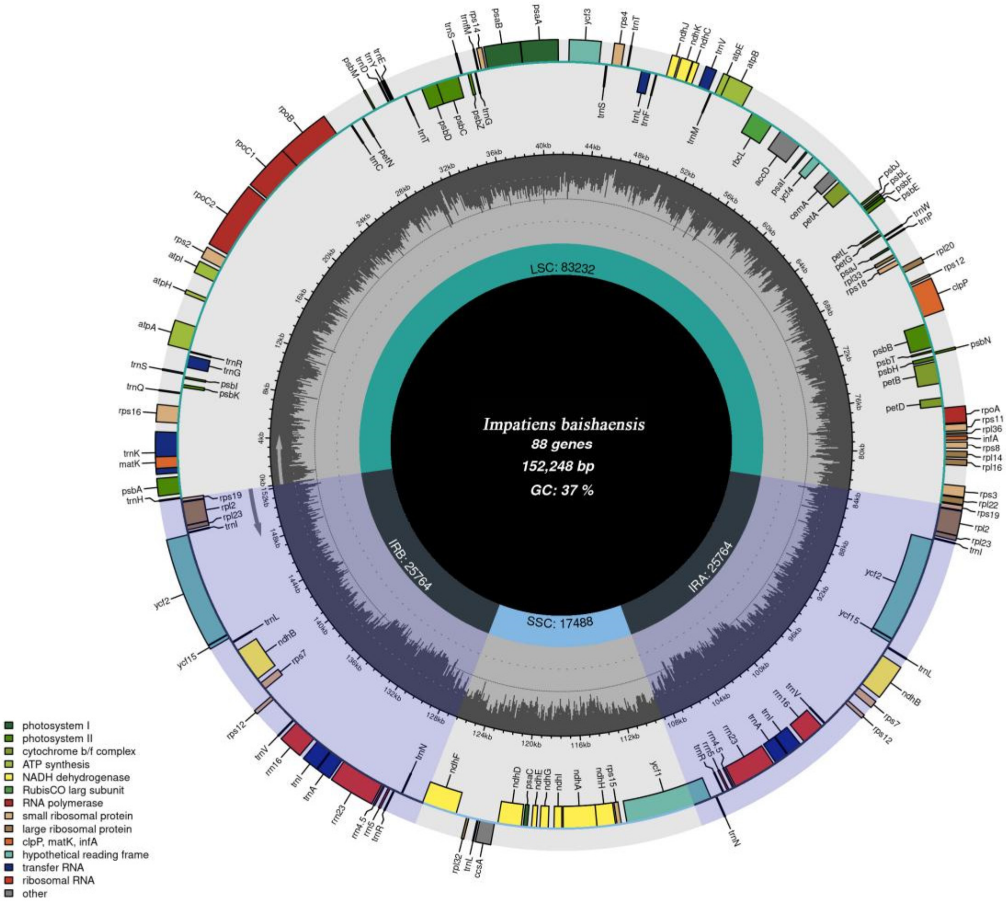

Fig. S2. Gene map of the *I. baishaensis* chloroplast genome. Genes transcribed clockwise are shown on the outside of the outer circle, while genes transcribed counterclockwise are located inside the inner circle. Genes with different functional groups are distinguished by colour coding.The positions of the long single-copy (LSC), short single-copy (SSC), and inverted repeat regions are shown in the inner circles

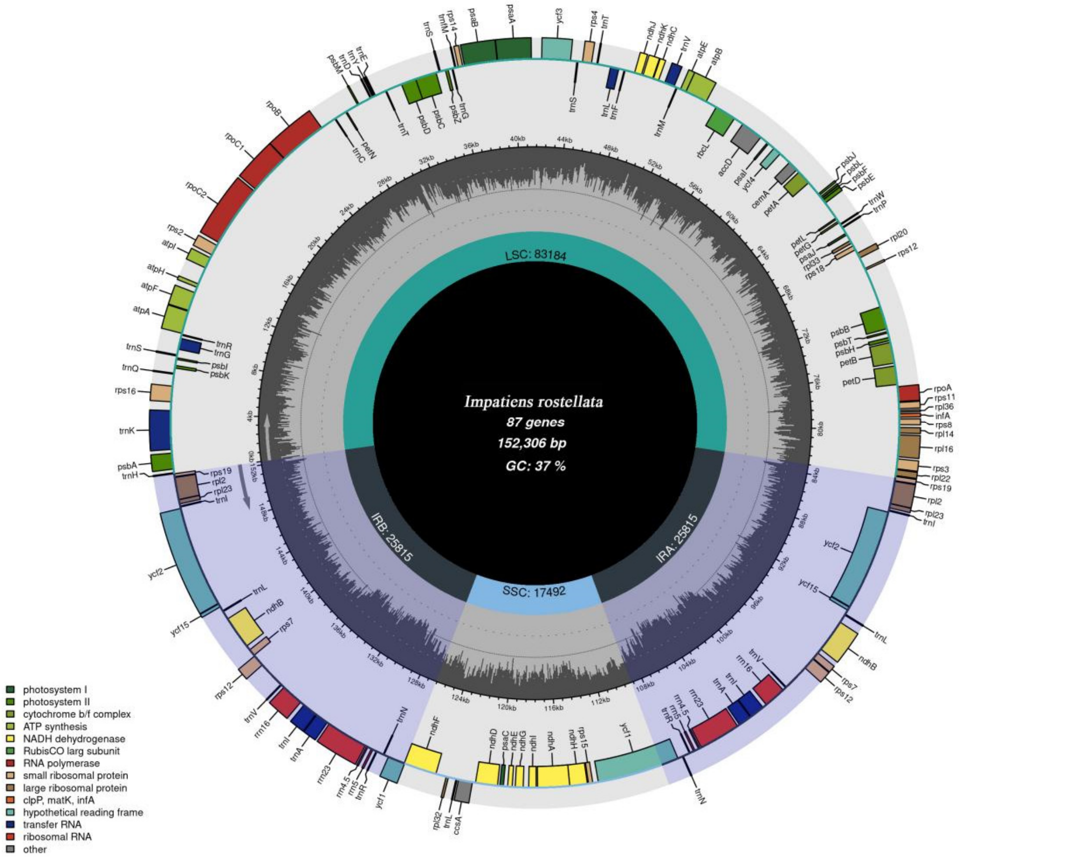

Fig. S3. Gene map of the *I. rostellata* chloroplast genome. Genes transcribed clockwise are shown on the outside of the outer circle, while genes transcribed counterclockwise are located inside the inner circle. Genes with different functional groups are distinguished by colour coding.The positions of the long single-copy (LSC), short single-copy (SSC), and inverted repeat regions are shown in the inner circles

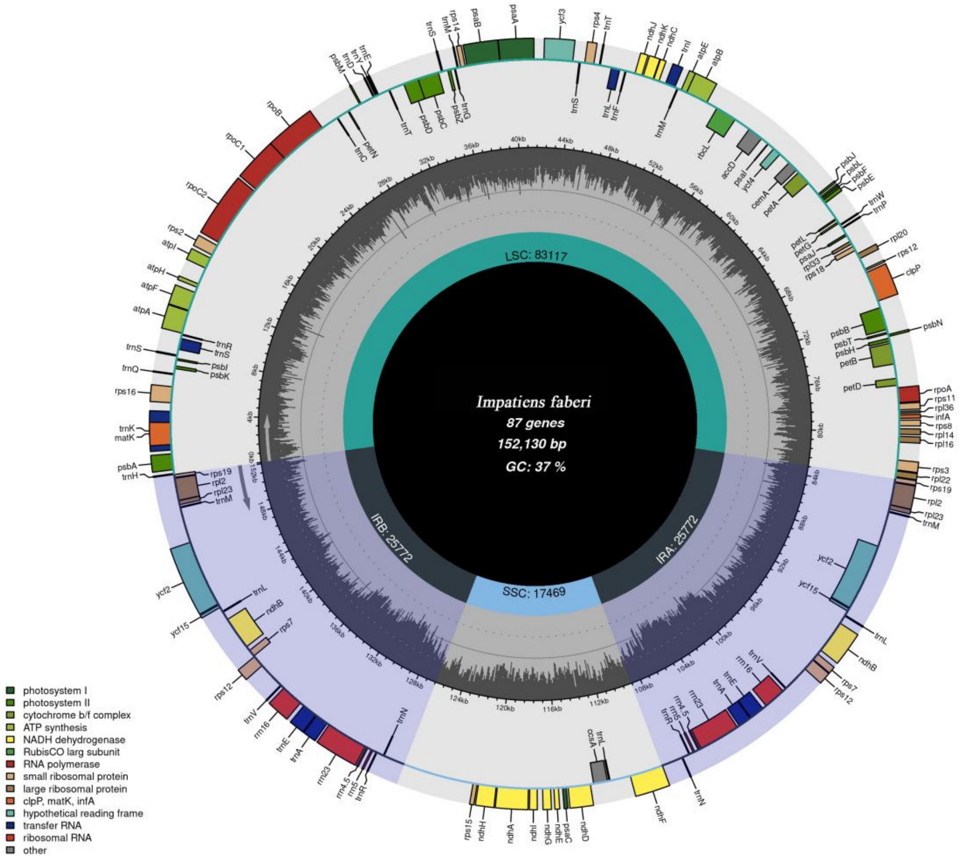

Fig. S4. Gene map of the *I. faberi* chloroplast genome. Genes transcribed clockwise are shown on the outside of the outer circle, while genes transcribed counterclockwise are located inside the inner circle. Genes with different functional groups are distinguished by colour coding.The positions of the long single-copy (LSC), short single-copy (SSC), and inverted repeat regions are shown in the inner circles

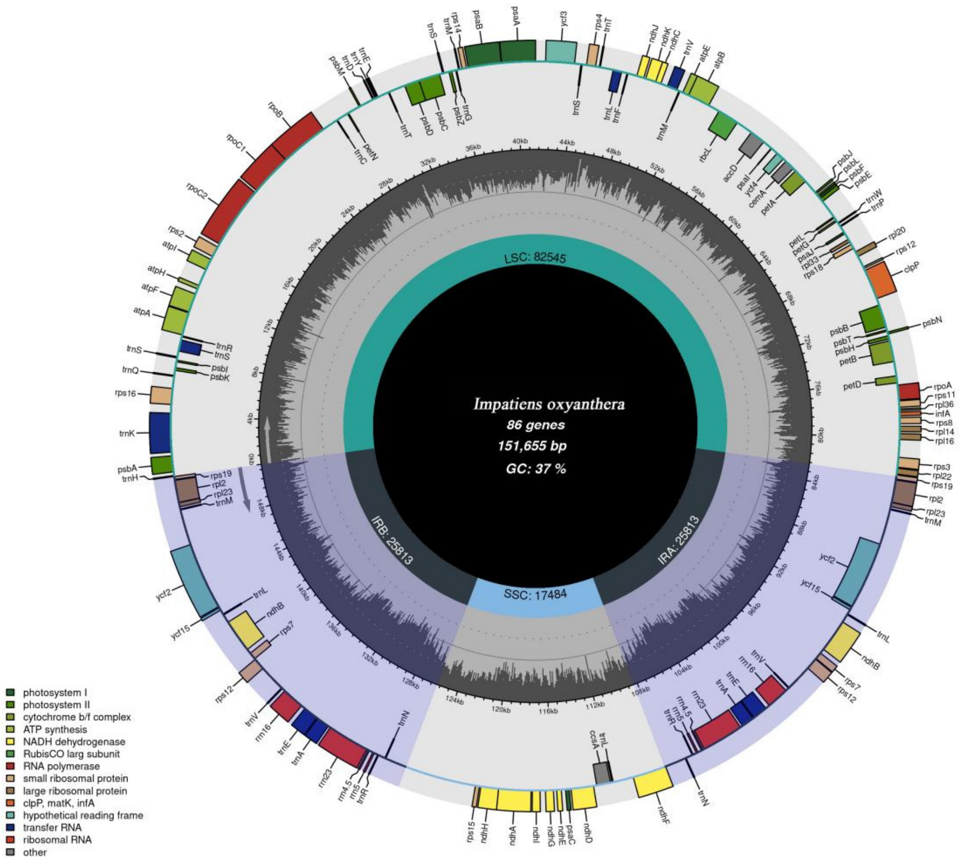

Fig. S5. Gene map of the *I. oxyanthera* chloroplast genome. Genes transcribed clockwise are shown on the outside of the outer circle, while genes transcribed counterclockwise are located inside the inner circle. Genes with different functional groups are distinguished by colour coding.The positions of the long single-copy (LSC), short single-copy (SSC), and inverted repeat regions are shown in the inner circles

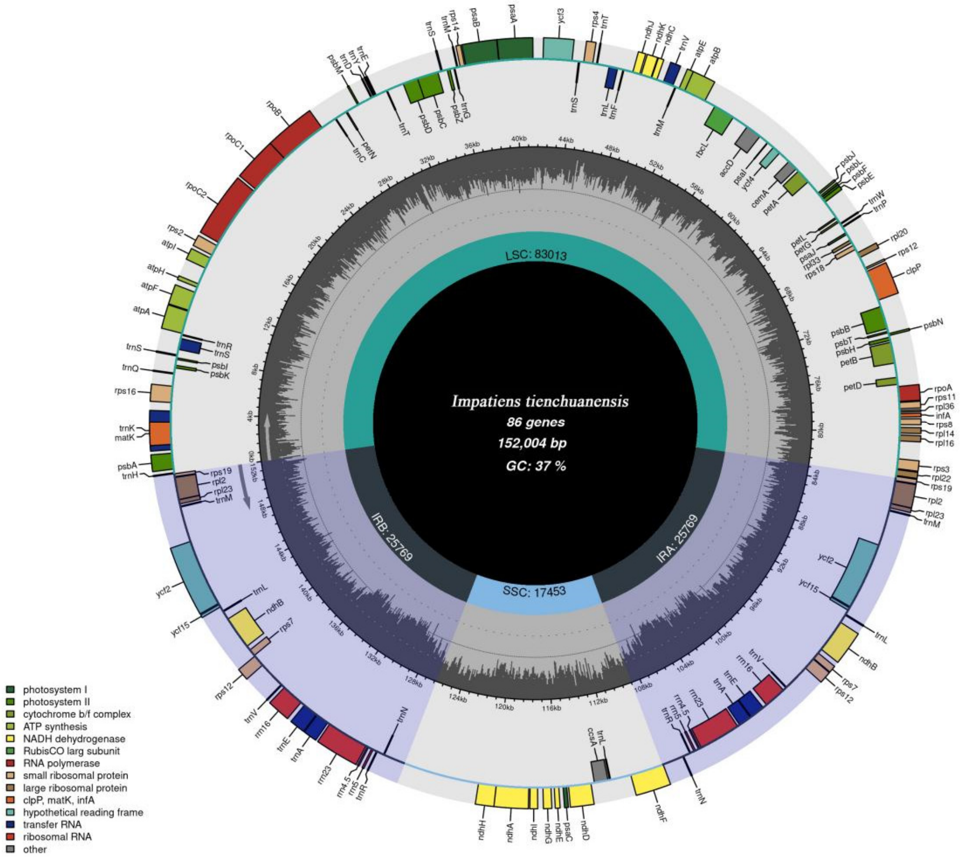

Fig. S6. Gene map of the *I. tienchuanensis* chloroplast genome. Genes transcribed clockwise are shown on the outside of the outer circle, while genes transcribed counterclockwise are located inside the inner circle. Genes with different functional groups are distinguished by colour coding.The positions of the long single-copy (LSC), short single-copy (SSC), and inverted repeat regions are shown in the inner circles

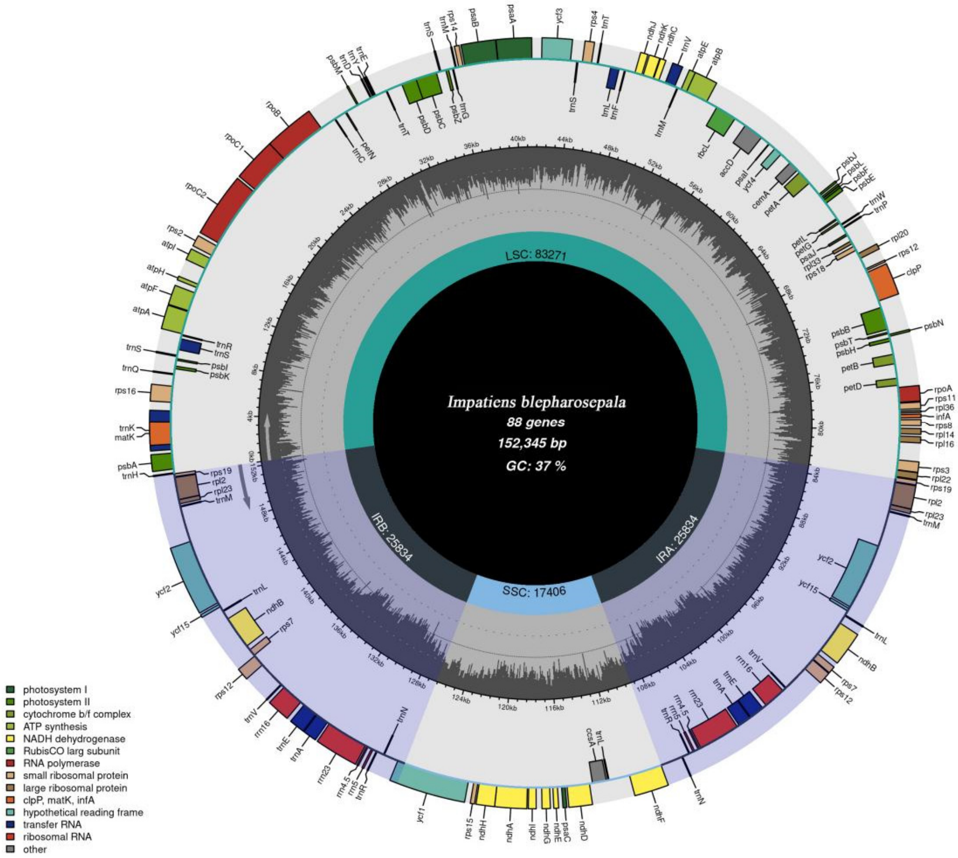

Fig. S7. Gene map of the *I. blepharosepala* chloroplast genome. Genes transcribed clockwise are shown on the outside of the outer circle, while genes transcribed counterclockwise are located inside the inner circle. Genes with different functional groups are distinguished by colour coding.The positions of the long single-copy (LSC), short single-copy (SSC), and inverted repeat regions are shown in the inner circles

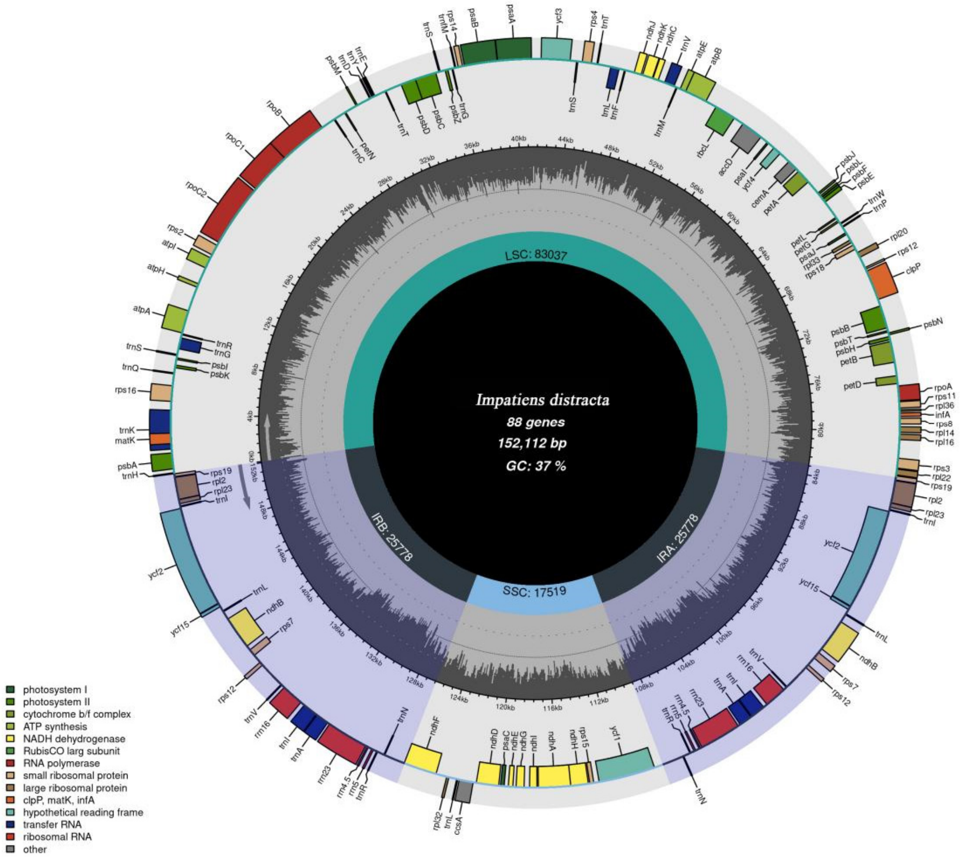

Fig. S8. Gene map of the *I. distracta* chloroplast genome. Genes transcribed clockwise are shown on the outside of the outer circle, while genes transcribed counterclockwise are located inside the inner circle. Genes with different functional groups are distinguished by colour coding.The positions of the long single-copy (LSC), short single-copy (SSC), and inverted repeat regions are shown in the inner circles

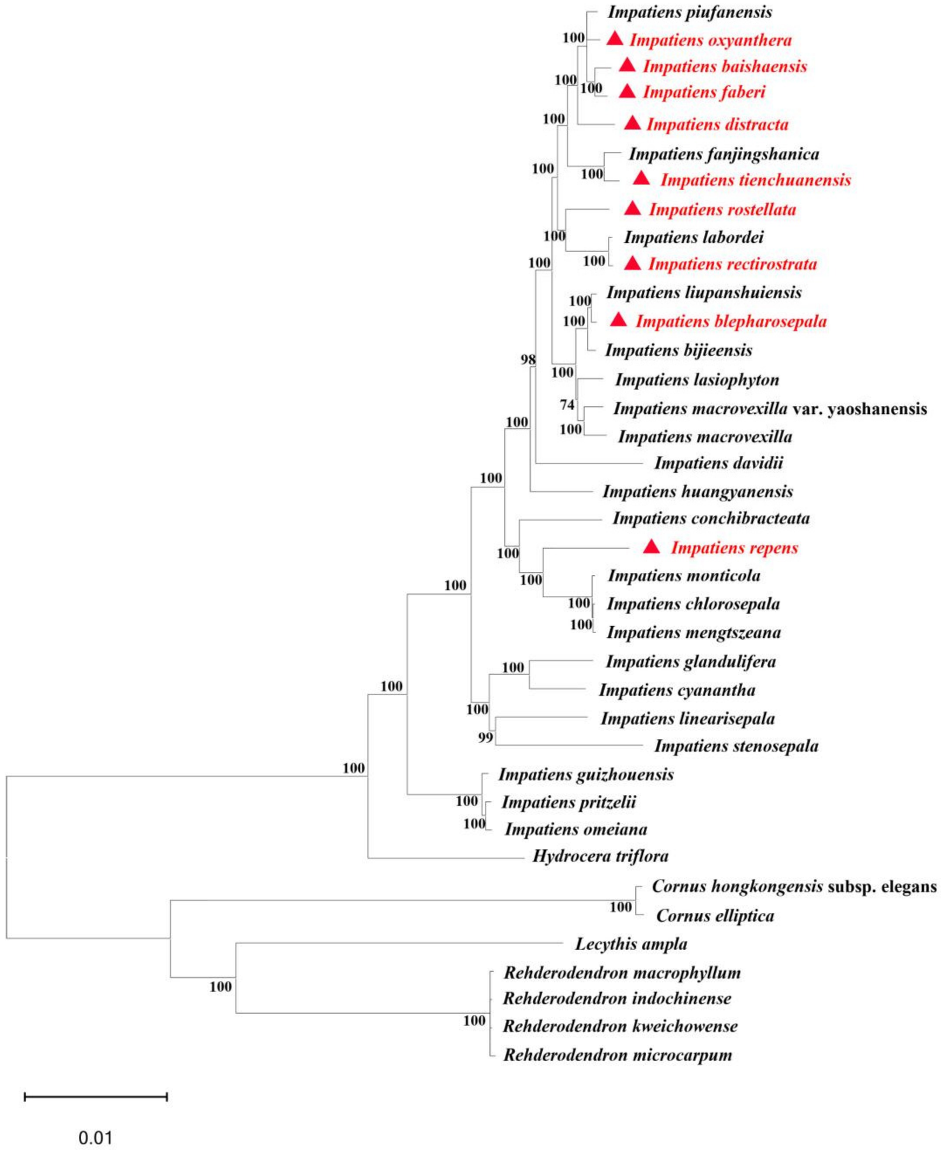

Fig. S9. Phylogenetic tree based on chloroplast genome sequences of 31 Bromeliaceae species and 7 other related species using the ML method. Text in bold red preceded by red triangles indicates the 9 *Impatiens* species studied here.

Table S1: Genes in the *I. rectirostrata* chloroplast genomes

Category	Gene group	Gene name
Photosynthesis	Subunits of photosystem I	*psaA*, *psaB*, *psaC*, *psaI*, *psaJ*
	Subunits of photosystem II	*psbA*, *psbB*, *psbC*(2), *psbD*, *psbE*, *psbF*, *psbH*, *psbI*, *psbJ*, *psbK*, *psbL*, *psbM*, *psbT*, *psbZ*
	Subunits of NADH dehydrogenase	*ndhA**(2), *ndhB**(2), *ndhC*, *ndhD*, *ndhE*, *ndhF*, *ndhG*, *ndhH*, *ndhI*, *ndhJ*, *ndhK*
	Subunits of cytochrome b/f complex	*petA*, *petB**, *petD*, *petD**, *petG*, *petL*, *petN*
	Subunits of ATP synthase	*atpA*, *atpB*, *atpE*, *atpF**(2), *atpH*, *atpI*
	Large subunit of rubisco	*rbcL*
	Subunits photochlorophyllide reductase	-
Self-replication	Proteins of large ribosomal subunit	*rpl14*, *rpl16**, *rpl2**(4), *rpl20*, *rpl22*, *rpl23*(2), *rpl32*, *rpl33*, *rpl36*
	Proteins of small ribosomal subunit	*rps11*, *rps12***(4), *rps14*, *rps15*, *rps16**(2), *rps18*, *rps19*(2), *rps2*, *rps3*, *rps4*, *rps7*(2), *rps8*
	Subunits of RNA polymerase	*rpoA*, *rpoB*, *rpoC1**(2), *rpoC2*(2)
	Ribosomal RNAs	*rrn16*(4), *rrn23*(4), *rrn4.5*(2), *rrn5*(2)
	Transfer RNAs	*trnA-UGC**(2), *trnC-GCA*(2), *trnD-GUC*, *trnE-UUC*, *trnF-GAA*, *trnG-GCC* | *trnG-GCC-fragment*, *trnG-GCC**, *trnG-UCC**, *trnH-GUG*(2), *trnI-CAU*(2), *trnI-GAU**(4), *trnK-UUU**(2), *trnL-CAA*(2), *trnL-UAA**(2), *trnL-UAG*, *trnM-CAU*(2), *trnN-GUU*(4), *trnP-GGG*, *trnP-UGG*, *trnQ-UUG*, *trnR-ACG*(2), *trnR-UCU*, *trnS-GCU*, *trnS-GGA*, *trnS-UGA*, *trnT-GGU*, *trnT-UGU*, *trnV-GAC*(2), *trnV-UAC*, *trnV-UAC**, *trnW-CCA*, *trnY-GUA*, *trnfM-CAU*
Other genes	Maturase	*matK*
	Protease	*clpP1***(2)
	Envelope membrane protein	*cemA*
	Acetyl-CoA carboxylase	*accD*
	c-type cytochrome synthesis gene	*ccsA*
	Translation initiation factor	*infA*
	other	*pafI***(2), *pafII*, *pbf1*
Genes of unknown function	Conserved hypothetical chloroplast ORF	*orf188*, *ycf1*(2), *ycf15*(2), *ycf2*(4)

Notes: Gene*: Gene with one intron; Gene**: Gene with two introns; Gene(2): Number of copies of multi-copy genes; Gene(4): Number of copies of multi-copy genes.

Table S2: Genes in the *I. baishaensis* chloroplast genomes

Category	Gene group	Gene name
Photosynthesis	Subunits of photosystem I	*psaA*, *psaB*, *psaC*, *psaI*, *psaJ*
	Subunits of photosystem II	*psbA*, *psbB*, *psbC*, *psbD*, *psbE*, *psbF*, *psbH*, *psbI*, *psbJ*, *psbK*, *psbL*, *psbM*, *psbN*, *psbT*, *psbZ*
	Subunits of NADH dehydrogenase	*ndhA**, *ndhB**(2), *ndhC*, *ndhD*, *ndhE*, *ndhF*, *ndhG*, *ndhH*, *ndhI*, *ndhJ*, *ndhK*
	Subunits of cytochrome b/f complex	*petA*, *petB**, *petD*, *petG*, *petL*, *petN*
	Subunits of ATP synthase	*atpA*, *atpB*, *atpE*, *atpH*, *atpI*
	Large subunit of rubisco	*rbcL*
	Subunits photochlorophyllide reductase	-
Self-replication	Proteins of large ribosomal subunit	*rpl14*, *rpl16*, *rpl2**(2), *rpl20*, *rpl22*, *rpl23*(2), *rpl32*, *rpl33*, *rpl36*
	Proteins of small ribosomal subunit	*rps11*, *rps12**(2), *rps14*, *rps15*, *rps16**, *rps18*, *rps19*(2), *rps2*, *rps3*, *rps4*, *rps7*(2), *rps8*
	Subunits of RNA polymerase	*rpoA*, *rpoB*, *rpoC1**, *rpoC2*
	Ribosomal RNAs	*rrn16S*(2), *rrn23S*(2), *rrn4.5S*(2), *rrn5S*(2)
	Transfer RNAs	*trnA-UGC**(2), *trnC-GCA*, *trnD-GUC*, *trnE-UUC*, *trnF-GAA*, *trnG-GCC*, *trnG-GCC**, *trnH-GUG*, *trnI-CAU*(2), *trnI-GAU**(2), *trnK-UUU**, *trnL-CAA*(2), *trnL-UAA**, *trnL-UAG*, *trnM-CAU*, *trnN-GUU*(2), *trnP-UGG*, *trnQ-UUG*, *trnR-ACG*(2), *trnR-UCU*, *trnS-GCU*(2), *trnS-UGA*, *trnT-GGU*, *trnT-UGU*, *trnV-GAC*(2), *trnV-UAC**, *trnW-CCA*, *trnY-GUA*, *trnfM-CAU*
Other genes	Maturase	*matK*
	Protease	*clpP***
	Envelope membrane protein	*cemA*
	Acetyl-CoA carboxylase	*accD*
	c-type cytochrome synthesis gene	*ccsA*
	Translation initiation factor	*infA*
	other	-
Genes of unknown function	Conserved hypothetical chloroplast ORF	*ycf1*, *ycf15*(2), *ycf2*(2), *ycf3***, *ycf4*

Notes: Gene*: Gene with one intron; Gene**: Gene with two introns; Gene(2): Number of copies of multi-copy genes; Gene(4): Number of copies of multi-copy genes.

Table S3: Genes in the *I. rostellata* chloroplast genomes

Category	Gene group	Gene name
Photosynthesis	Subunits of photosystem I	*psaA*, *psaB*, *psaC*, *psaI*, *psaJ*
	Subunits of photosystem II	*psbA*, *psbB*, *psbC*, *psbD*, *psbE*, *psbF*, *psbH*, *psbI*, *psbJ*, *psbK*, *psbL*, *psbM*, *psbT*, *psbZ*
	Subunits of NADH dehydrogenase	*ndhA**(2), *ndhB**(2), *ndhC*, *ndhD*, *ndhE*, *ndhF*, *ndhG*, *ndhH*, *ndhI*, *ndhJ*, *ndhK*
	Subunits of cytochrome b/f complex	*petA*, *petB**, *petD**, *petG*, *petL*, *petN*
	Subunits of ATP synthase	*atpA*, *atpB*, *atpE*, *atpF**(2), *atpH*, *atpI*
	Large subunit of rubisco	*rbcL*
	Subunits photochlorophyllide reductase	-
Self-replication	Proteins of large ribosomal subunit	*rpl14*, *rpl16**, *rpl2**(4), *rpl20*, *rpl22*, *rpl23*(2), *rpl32*, *rpl33*, *rpl36*
	Proteins of small ribosomal subunit	*rps11*, *rps12***(2), *rps14*, *rps15*, *rps16**, *rps18*, *rps19*(2), *rps2*, *rps3*, *rps4*, *rps7*(2), *rps8*
	Subunits of RNA polymerase	*rpoA*, *rpoB*, *rpoC1**, *rpoC2*
	Ribosomal RNAs	*rrn16*(4), *rrn23*(4), *rrn4.5*(2), *rrn5*(2)
	Transfer RNAs	*trnA-UGC**(2), *trnC-GCA*, *trnD-GUC*, *trnE-UUC*, *trnF-GAA*, *trnG-GCC*, *trnG-UCC**, *trnH-GUG*, *trnI-CAU*(2), *trnI-GAU**(4), *trnK-UUU**, *trnL-CAA*(2), *trnL-UAA**, *trnL-UAG*, *trnM-CAU*, *trnN-GUU*(4), *trnP-UGG*, *trnQ-UUG*, *trnR-ACG*(2), *trnR-UCU*, *trnS-GCU*, *trnS-GGA*, *trnS-UGA*, *trnT-GGU*, *trnT-UGU*, *trnV-GAC*(2), *trnV-UAC**, *trnW-CCA*, *trnY-GUA*, *trnfM-CAU*
Other genes	Maturase	*matK*
	Protease	*clpP1***
	Envelope membrane protein	*cemA*
	Acetyl-CoA carboxylase	*accD*
	c-type cytochrome synthesis gene	*ccsA*
	Translation initiation factor	*infA*
	other	*pafI***(2), *pafII*, *pbf1*
Genes of unknown function	Conserved hypothetical chloroplast ORF	*ycf1*(2), *ycf2*(4)

Notes: Gene*: Gene with one intron; Gene**: Gene with two introns; Gene(2): Number of copies of multi-copy genes; Gene(4): Number of copies of multi-copy genes.

Table S4: Genes in the *I. faberi* chloroplast genomes

Category	Gene group	Gene name
Photosynthesis	Subunits of photosystem I	*psaA*, *psaB*, *psaC*, *psaI*, *psaJ*
	Subunits of photosystem II	*psbA*, *psbB*, *psbC*, *psbD*, *psbE*, *psbF*, *psbH*, *psbI*, *psbJ*, *psbK*, *psbL*, *psbM*, *psbN*, *psbT*, *psbZ*
	Subunits of NADH dehydrogenase	*ndhA**, *ndhB**(2), *ndhC*, *ndhD*, *ndhE*, *ndhF*, *ndhG*, *ndhH*, *ndhI*, *ndhJ*, *ndhK*
	Subunits of cytochrome b/f complex	*petA*, *petB**, *petD*, *petG*, *petL*, *petN*
	Subunits of ATP synthase	*atpA*, *atpB*, *atpE*, *atpF**, *atpH*, *atpI*
	Large subunit of rubisco	*rbcL*
	Subunits photochlorophyllide reductase	-
Self-replicatio n	Proteins of large ribosomal subunit	*rpl14*, *rpl16*, *rpl2**(2), *rpl20*, *rpl22*, *rpl23*(2), *rpl33*, *rpl36*
	Proteins of small ribosomal subunit	*rps11*, *rps12***(2), *rps14*, *rps15*, *rps16**, *rps18*, *rps19*(2), *rps2*, *rps3*, *rps4*, *rps7*(2), *rps8*
	Subunits of RNA polymerase	*rpoA*, *rpoB*, *rpoC1**, *rpoC2*
	Ribosomal RNAs	*rrn16S*(2), *rrn23S*(2), *rrn4.5S*(2), *rrn5S*(2)
	Transfer RNAs	*trnA-UGC**(2), *trnC-GCA*, *trnD-GUC*, *trnE-UUC*, *trnE-UUC**(2), *trnF-GAA*, *trnG-GCC*, *trnH-GUG*, *trnI-AAU**, *trnK-UUU**, *trnL-CAA*(2), *trnL-UAA**, *trnL-UAG*, *trnM-CAU*(4), *trnN-GUU*(2), *trnP-UGG*, *trnQ-UUG*, *trnR-ACG*(2), *trnR-UCU*, *trnS-CGA**, *trnS-GCU*, *trnS-GGA*, *trnS-UGA*, *trnT-GGU*, *trnT-UGU*, *trnV-GAC*(2), *trnW-CCA*, *trnY-GUA*
Other genes	Maturase	*matK*
	Protease	*clpP***
	Envelope membrane protein	*cemA*
	Acetyl-CoA carboxylase	*accD*
	c-type cytochrome synthesis gene	*ccsA*
	Translation initiation factor	*infA*
	other	-
Genes of unknown function	Conserved hypothetical chloroplast ORF	*ycf15*(2), *ycf2*(2), *ycf3***, *ycf4*

Notes: Gene*: Gene with one intron; Gene**: Gene with two introns; Gene(2): Number of copies of multi-copy genes; Gene(4): Number of copies of multi-copy genes.

Table S5: Genes in the *I. oxyanthera var.* chloroplast genomes

Category	Gene group	Gene name
Photosynthesis	Subunits of photosystem I	*psaA*, *psaB*, *psaC*, *psaI*, *psaJ*
	Subunits of photosystem II	*psbA*, *psbB*, *psbC*, *psbD*, *psbE*, *psbF*, *psbH*, *psbI*, *psbJ*, *psbK*, *psbL*, *psbM*, *psbN*, *psbT*, *psbZ*
	Subunits of NADH dehydrogenase	*ndhA**, *ndhB**(2), *ndhC*, *ndhD*, *ndhE*, *ndhF*, *ndhG*, *ndhH*, *ndhI*, *ndhJ*, *ndhK*
	Subunits of cytochrome b/f complex	*petA*, *petB**, *petD*, *petG*, *petL*, *petN*
	Subunits of ATP synthase	*atpA*, *atpB*, *atpE*, *atpF**, *atpH*, *atpI*
	Large subunit of rubisco	*rbcL*
	Subunits photochlorophyllide reductase	-
Self-replication	Proteins of large ribosomal subunit	*rpl14*, *rpl16*, *rpl2**(2), *rpl20*, *rpl22*, *rpl23*(2), *rpl33*, *rpl36*
	Proteins of small ribosomal subunit	*rps11*, *rps12***(2), *rps14*, *rps15*, *rps16**, *rps18*, *rps19*(2), *rps2*, *rps3*, *rps4*, *rps7*(2), *rps8*
	Subunits of RNA polymerase	*rpoA*, *rpoB*, *rpoC1**, *rpoC2*
	Ribosomal RNAs	*rrn16S*(2), *rrn23S*(2), *rrn4.5S*(2), *rrn5S*(2)
	Transfer RNAs	*trnA-UGC**(2), *trnC-GCA*, *trnD-GUC*, *trnE-UUC*, *trnE-UUC**(2), *trnF-GAA*, *trnG-GCC*, *trnH-GUG*, *trnK-UUU**, *trnL-CAA*(2), *trnL-UAA**, *trnL-UAG*, *trnM-CAU*(4), *trnN-GUU*(2), *trnP-UGG*, *trnQ-UUG*, *trnR-ACG*(2), *trnR-UCU*, *trnS-CGA**, *trnS-GCU*, *trnS-GGA*, *trnS-UGA*, *trnT-GGU*, *trnT-UGU*, *trnV-GAC*(2), *trnV-UAC**, *trnW-CCA*, *trnY-GUA*
Other genes	Maturase	-
	Protease	*clpP***
	Envelope membrane protein	*cemA*
	Acetyl-CoA carboxylase	*accD*
	c-type cytochrome synthesis gene	*ccsA*
	Translation initiation factor	*infA*
	other	-
Genes of unknown function	Conserved hypothetical chloroplast ORF	*ycf15*(2), *ycf2**(2), *ycf3***, *ycf4*

Notes: Gene*: Gene with one intron; Gene**: Gene with two introns; Gene(2): Number of copies of multi-copy genes; Gene(4): Number of copies of multi-copy genes.

Table S6: Genes in the *I. tienchuanensis* chloroplast genomes

Category	Gene group	Gene name
Photosynthesis	Subunits of photosystem I	*psaA*, *psaB*, *psaC*, *psaI*, *psaJ*
	Subunits of photosystem II	*psbA*, *psbB*, *psbC*, *psbD*, *psbE*, *psbF*, *psbH*, *psbI*, *psbJ*, *psbK*, *psbL*, *psbM*, *psbN*, *psbT*, *psbZ*
	Subunits of NADH dehydrogenase	*ndhA**, *ndhB**(2), *ndhC*, *ndhD*, *ndhE*, *ndhF*, *ndhG*, *ndhH*, *ndhI*, *ndhJ*, *ndhK*
	Subunits of cytochrome b/f complex	*petA*, *petB**, *petD*, *petG*, *petL*, *petN*
	Subunits of ATP synthase	*atpA*, *atpB*, *atpE*, *atpF**, *atpH*, *atpI*
	Large subunit of rubisco	*rbcL*
	Subunits photochlorophyllide reductase	-
Self-replication	Proteins of large ribosomal subunit	*rpl14*, *rpl16*, *rpl2**(2), *rpl20*, *rpl22*, *rpl23*(2), *rpl33*, *rpl36*
	Proteins of small ribosomal subunit	*rps11*, *rps12***(2), *rps14*, *rps16**, *rps18*, *rps19*(2), *rps2*, *rps3*, *rps4*, *rps7*(2), *rps8*
	Subunits of RNA polymerase	*rpoA*, *rpoB*, *rpoC1**, *rpoC2*
	Ribosomal RNAs	*rrn16S*(2), *rrn23S*(2), *rrn4.5S*(2), *rrn5S*(2)
	Transfer RNAs	*trnA-UGC**(2), *trnC-GCA*, *trnD-GUC*, *trnE-UUC*, *trnE-UUC**(2), *trnF-GAA*, *trnG-GCC*, *trnH-GUG*, *trnK-UUU**, *trnL-CAA*(2), *trnL-UAA**, *trnL-UAG*, *trnM-CAU*(4), *trnN-GUU*(2), *trnP-UGG*, *trnQ-UUG*, *trnR-ACG*(2), *trnR-UCU*, *trnS-CGA**, *trnS-GCU*, *trnS-GGA*, *trnS-UGA*, *trnT-GGU*, *trnT-UGU*, *trnV-GAC*(2), *trnV-UAC**, *trnW-CCA*, *trnY-GUA*
Other genes	Maturase	*matK*
	Protease	*clpP***
	Envelope membrane protein	*cemA*
	Acetyl-CoA carboxylase	*accD*
	c-type cytochrome synthesis gene	*ccsA*
	Translation initiation factor	*infA*
	other	-
Genes of unknown function	Conserved hypothetical chloroplast ORF	*ycf15*(2), *ycf2**(2), *ycf3***, *ycf4*

Notes: Gene*: Gene with one intron; Gene**: Gene with two introns; Gene(2): Number of copies of multi-copy genes; Gene(4): Number of copies of multi-copy genes.

Table S7: Genes in the *I. blepharosepala* chloroplast genomes

Category	Gene group	Gene name
	Subunits of photosystem I	*psaA*, *psaB*, *psaC*, *psaI*, *psaJ*
	Subunits of photosystem II	*psbA*, *psbB*, *psbC*, *psbD*, *psbE*, *psbF*, *psbH*, *psbI*, *psbJ*, *psbK*, *psbL*, *psbM*, *psbN*, *psbT*, *psbZ*
	Subunits of NADH dehydrogenase	*ndhA**, *ndhB**(2), *ndhC*, *ndhD*, *ndhE*, *ndhF*, *ndhG*, *ndhH*, *ndhI*, *ndhJ*, *ndhK*
Photosynthesis	Subunits of cytochrome b/f complex	*petA*, *petB*, *petD*, *petG*, *petL*, *petN*
	Subunits of ATP synthase	*atpA*, *atpB*, *atpE*, *atpF**, *atpH*, *atpI*
	Large subunit of rubisco	*rbcL*
	Subunits photochlorophyllide reductase	-
	Proteins of large ribosomal subunit	*rpl14*, *rpl16*, *rpl2**(2), *rpl20*, *rpl22*, *rpl23*(2), *rpl33*, *rpl36*
	Proteins of small ribosomal subunit	*rps11*, *rps12***(2), *rps14*, *rps15*, *rps16**, *rps18*, *rps19*(2), *rps2*, *rps3*, *rps4*, *rps7*(2), *rps8*
Self-replication	Subunits of RNA polymerase	*rpoA*, *rpoB*, *rpoC1**, *rpoC2*
	Ribosomal RNAs	*rrn16S*(2), *rrn23S*(2), *rrn4.5S*(2), *rrn5S*(2)
	Transfer RNAs	*trnA-UGC**(2), *trnC-GCA*, *trnD-GUC*, *trnE-UUC*, *trnE-UUC**(2), *trnF-GAA*, *trnG-GCC*, *trnH-GUG*, *trnK-UUU**, *trnL-CAA*(2), *trnL-UAA**, *trnL-UAG*, *trnM-CAU*(4), *trnN-GUU*(2), *trnP-UGG*, *trnQ-UUG*, *trnR-ACG*(2), *trnR-UCU*, *trnS-CGA**, *trnS-GCU*, *trnS-GGA*, *trnS-UGA*, *trnT-GGU*, *trnT-UGU*, *trnV-GAC*(2), *trnV-UAC**, *trnW-CCA*, *trnY-GUA*
	Maturase	*matK*
	Protease	*clpP***
	Envelope membrane protein	*cemA*
Other genes	Acetyl-CoA carboxylase	*accD*
	c-type cytochrome synthesis gene	*ccsA*
	Translation initiation factor	*infA*
	other	-
Genes of unknown function	Conserved hypothetical chloroplast ORF	*ycf1*, *ycf15*(2), *ycf2*(2), *ycf3***, *ycf4*

Notes: Gene*: Gene with one intron; Gene**: Gene with two introns; Gene(2): Number of copies of multi-copy genes; Gene(4): Number of copies of multi-copy genes.

Table S8: Genes in the *I. distracta* chloroplast genomes

Category	Gene group	Gene name
Photosynthesis	Subunits of photosystem I	*psaA*, *psaB*, *psaC*, *psaI*, *psaJ*
	Subunits of photosystem II	*psbA*, *psbB*, *psbC*(2), *psbD*, *psbE*, *psbF*, *psbH*, *psbI*, *psbJ*, *psbK*, *psbL*, *psbM*, *psbT*, *psbZ*
	Subunits of NADH dehydrogenase	*ndhA**(2), *ndhB**(2), *ndhC*, *ndhD*, *ndhE*, *ndhF*, *ndhG*, *ndhH*, *ndhI*, *ndhJ*, *ndhK*
	Subunits of cytochrome b/f complex	*petA*, *petB**, *petD**, *petG*, *petL*, *petN*
	Subunits of ATP synthase	*atpA*, *atpB*, *atpE*, *atpF**(2), *atpH*, *atpI*
	Large subunit of rubisco	*rbcL*
	Subunits photochlorophyllide reductase	-
Self-replication	Proteins of large ribosomal subunit	*rpl14*, *rpl16**, *rpl2**(4), *rpl20*, *rpl22*, *rpl23*(2), *rpl32*, *rpl33*, *rpl36*
	Proteins of small ribosomal subunit	*rps11*, *rps12***(4), *rps14*, *rps15*, *rps16**(2), *rps18*, *rps19*(2), *rps2*, *rps3*, *rps4*, *rps7*(2), *rps8*
	Subunits of RNA polymerase	*rpoA*, *rpoB*, *rpoC1**(2), *rpoC2*(2)
	Ribosomal RNAs	*rrn16*(4), *rrn23*(4), *rrn4.5*(2), *rrn5*(2)
	Transfer RNAs	*trnA-UGC**(2), *trnC-GCA*(2), *trnD-GUC*, *trnE-UUC*, *trnF-GAA*, *trnG-GCC* | *trnG-GCC-fragment*, *trnG-GCC**, *trnG-UCC**, *trnH-GUG*(2), *trnI-CAU*(2), *trnI-GAU**(4), *trnK-UUU**(2), *trnL-CAA*(2), *trnL-UAA**(2), *trnL-UAG*, *trnM-CAU*(2), *trnN-GUU*(4), *trnP-GGG*, *trnP-UGG*, *trnQ-UUG*, *trnR-ACG*(2), *trnR-UCU*, *trnS-GCU*, *trnS-GGA*, *trnS-UGA*, *trnT-GGU*, *trnT-UGU*, *trnV-GAC*(2), *trnV-UAC*, *trnV-UAC**, *trnW-CCA*, *trnY-GUA*, *trnfM-CAU*
Other genes	Maturase	*matK*(2)
	Protease	*clpP1***(2)
	Envelope membrane protein	*cemA*
	Acetyl-CoA carboxylase	*accD*
	c-type cytochrome synthesis gene	*ccsA*
	Translation initiation factor	*infA*
	other	*pafI***(2), *pafII*, *pbf1*
Genes of unknown function	Conserved hypothetical chloroplast ORF	*orf188*, *ycf1*(2), *ycf15*(2), *ycf2*(4)

Notes: Gene*: Gene with one intron; Gene**: Gene with two introns; Gene(2): Number of copies of multi-copy genes; Gene(4): Number of copies of multi-copy genes.

Table S9: Codon usage and RSCU of *I. repens* chloroplast genome.

Codon	AA	RSCU	% of AA	Count	Codon	AA	RSCU	% of AA	Count
GCA	A	1.20	0.86%	436	CCA	P	1.30	1.51%	767
GCC	A	0.95	0.68%	345	CCC	P	1.01	1.18%	596
GCG	A	0.62	0.44%	225	CCG	P	0.65	0.76%	385
GCU	A	1.23	0.88%	447	CCU	P	1.03	1.20%	607
UGC	C	0.77	0.84%	424	CAA	Q	1.40	1.99%	1,008
UGU	C	1.23	1.34%	677	CAG	Q	0.60	0.85%	431
GAC	D	0.52	0.76%	386	AGA	R	2.03	2.07%	1,051
GAU	D	1.48	2.16%	1,093	AGG	R	1.21	1.23%	624
GAA	E	1.37	2.51%	1,272	CGA	R	1.00	1.02%	518
GAG	E	0.63	1.14%	579	CGC	R	0.44	0.45%	227
UUC	F	0.74	2.77%	1,403	CGG	R	0.64	0.65%	329
UUU	F	1.26	4.71%	2,384	CGU	R	0.69	0.70%	356
GGA	G	1.41	1.44%	729	AGC	S	0.61	0.94%	476
GGC	G	0.62	0.63%	318	AGU	S	0.88	1.34%	679
GGG	G	1.02	1.03%	524	UCA	S	1.19	1.81%	918
GGU	G	0.95	0.97%	492	UCC	S	1.10	1.69%	856
CAC	H	0.57	0.74%	376	UCG	S	0.73	1.12%	566
CAU	H	1.43	1.88%	950	UCU	S	1.49	2.28%	1,153
AUA	I	1.04	2.89%	1,462	ACA	T	1.13	1.22%	618
AUC	I	0.69	1.90%	965	ACC	T	1.03	1.11%	563
AUU	I	1.28	3.54%	1,792	ACG	T	0.64	0.68%	346
AAA	K	1.40	4.50%	2,282	ACU	T	1.20	1.29%	653
AAG	K	0.60	1.95%	986	GUA	V	1.18	1.29%	656
CUA	L	0.91	1.52%	769	GUC	V	0.73	0.80%	407
CUC	L	0.73	1.22%	618	GUG	V	0.76	0.83%	421
CUG	L	0.60	1.01%	510	GUU	V	1.33	1.46%	740
CUU	L	1.26	2.10%	1,066	UGG	W	1.00	1.40%	707
UUA	L	1.24	2.07%	1,049	UAC	Y	0.62	1.25%	634
UUG	L	1.26	2.10%	1,066	UAU	Y	1.38	2.81%	1,423
AUG	M	1.00	1.60%	811	UAA	*	1.24	2.39%	1,211
AAC	N	0.59	1.51%	763	UAG	*	0.80	1.53%	776
AAU	N	1.41	3.59%	1,818	UGA	*	0.97	1.87%	946

Table S10: Codon usage and RSCU of *I. rectirostrata* chloroplast genome.

Codon	AA	RSCU	% of AA	Count	Codon	AA	RSCU	% of AA	Count
GCA	A	1.23	0.96%	483	CCA	P	1.24	1.48%	742
GCC	A	0.88	0.68%	343	CCC	P	1.00	1.19%	598
GCG	A	0.52	0.41%	206	CCG	P	0.58	0.70%	350
GCT	A	1.37	1.07%	536	CCT	P	1.18	1.41%	708
TGC	C	0.78	0.84%	424	CAA	Q	1.40	1.87%	942
TGT	C	1.22	1.31%	659	CAG	Q	0.60	0.80%	404
GAC	D	0.54	0.81%	409	AGA	R	2.04	2.09%	1,053
GAT	D	1.46	2.18%	1,095	AGG	R	1.09	1.11%	560
GAA	E	1.40	2.60%	1,306	CGA	R	1.03	1.05%	529
GAG	E	0.60	1.12%	561	CGC	R	0.48	0.50%	249
TTC	F	0.73	2.63%	1,324	CGG	R	0.66	0.68%	341
TTT	F	1.27	4.60%	2,310	CGT	R	0.71	0.73%	368
GGA	G	1.46	1.56%	786	AGC	S	0.61	0.94%	474
GGC	G	0.59	0.64%	320	AGT	S	0.86	1.33%	670
GGG	G	0.94	1.01%	507	TCA	S	1.34	2.09%	1,052
GGT	G	1.01	1.08%	545	TCC	S	1.10	1.71%	860
CAC	H	0.61	0.75%	375	TCG	S	0.64	0.99%	500
CAT	H	1.39	1.69%	848	TCT	S	1.46	2.28%	1,144
ATA	I	1.01	2.85%	1,431	ACA	T	1.24	1.32%	664
ATC	I	0.72	2.02%	1,014	ACC	T	1.00	1.07%	540
ATT	I	1.27	3.55%	1,783	ACG	T	0.58	0.62%	312
AAA	K	1.39	4.38%	2,201	ACT	T	1.18	1.26%	634
AAG	K	0.61	1.91%	961	GTA	V	1.25	1.40%	704
CTA	L	0.93	1.56%	785	GTC	V	0.75	0.84%	423
CTC	L	0.71	1.19%	596	GTG	V	0.66	0.74%	370
CTG	L	0.52	0.87%	435	GTT	V	1.35	1.51%	760
CTT	L	1.26	2.12%	1,066	TGG	W	1.00	1.32%	666
TTA	L	1.42	2.38%	1,198	TAC	Y	0.65	1.23%	617
TTG	L	1.17	1.97%	988	TAT	Y	1.35	2.55%	1,281
ATG	M	1.00	1.66%	835	TAA	*	1.16	2.24%	1,127
AAC	N	0.59	1.48%	742	TAG	*	0.79	1.53%	768
AAT	N	1.41	3.53%	1,772	TGA	*	1.05	2.02%	1,016

Table S11: Codon usage and RSCU of *I. baishaensis* chloroplast genome.

Codon	AA	RSCU	% of AA	Count	Codon	AA	RSCU	% of AA	Count
GCA	A	1.18	0.85%	433	CCA	P	1.25	1.44%	731
GCC	A	0.94	0.68%	345	CCC	P	0.99	1.14%	578
GCG	A	0.60	0.43%	219	CCG	P	0.65	0.75%	379
GCT	A	1.28	0.92%	469	CCT	P	1.11	1.28%	649
TGC	C	0.78	0.78%	396	CAA	Q	1.35	2.10%	1,065
TGT	C	1.22	1.21%	616	CAG	Q	0.65	1.02%	518
GAC	D	0.55	0.83%	421	AGA	R	2.02	1.95%	992
GAT	D	1.45	2.17%	1,099	AGG	R	1.10	1.07%	541
GAA	E	1.35	2.54%	1,291	CGA	R	1.04	1.01%	512
GAG	E	0.65	1.22%	620	CGC	R	0.47	0.46%	232
TTC	F	0.74	2.65%	1,347	CGG	R	0.70	0.68%	346
TTT	F	1.26	4.57%	2,317	CGT	R	0.68	0.66%	333
GGA	G	1.46	1.54%	779	AGC	S	0.56	0.83%	421
GGC	G	0.58	0.61%	309	AGT	S	0.91	1.35%	684
GGG	G	1.00	1.06%	538	TCA	S	1.30	1.93%	979
GGT	G	0.96	1.01%	514	TCC	S	1.10	1.63%	829
CAC	H	0.59	0.75%	383	TCG	S	0.65	0.96%	486
CAT	H	1.41	1.81%	916	TCT	S	1.48	2.19%	1,112
ATA	I	1.04	2.85%	1,448	ACA	T	1.22	1.30%	660
ATC	I	0.68	1.86%	946	ACC	T	0.96	1.03%	523
ATT	I	1.28	3.50%	1,775	ACG	T	0.58	0.61%	312
AAA	K	1.34	4.33%	2,196	ACT	T	1.24	1.32%	671
AAG	K	0.66	2.13%	1,080	GTA	V	1.17	1.34%	681
CTA	L	0.94	1.65%	835	GTC	V	0.71	0.82%	415
CTC	L	0.74	1.30%	659	GTG	V	0.76	0.87%	444
CTG	L	0.54	0.94%	478	GTT	V	1.36	1.56%	790
CTT	L	1.22	2.14%	1,087	TGG	W	1.00	1.45%	735
TTA	L	1.31	2.29%	1,164	TAC	Y	0.65	1.34%	680
TTG	L	1.24	2.17%	1,099	TAT	Y	1.35	2.78%	1,412
ATG	M	1.00	1.70%	865	TAA	*	1.18	2.25%	1,140
AAC	N	0.59	1.44%	732	TAG	*	0.87	1.65%	839
AAT	N	1.41	3.48%	1,766	TGA	*	0.95	1.81%	918

Table S12: Codon usage and RSCU of *I. rostellata* chloroplast genome.

Codon	AA	RSCU	% of AA	Count	Codon	AA	RSCU	% of AA	Count
GCA	A	1.23	0.12%	493	CCA	P	1.27	0.13%	777
GCC	A	0.90	0.09%	362	CCC	P	1.00	0.10%	610
GCG	A	0.54	0.05%	216	CCG	P	0.58	0.06%	358
GCU	A	1.34	0.13%	537	CCU	P	1.16	0.12%	709
UGC	C	0.81	0.08%	431	CAA	Q	1.43	0.14%	1,093
UGU	C	1.19	0.12%	630	CAG	Q	0.57	0.06%	434
GAC	D	0.54	0.05%	409	AGA	R	1.98	0.20%	1,009
GAU	D	1.46	0.15%	1,116	AGG	R	1.09	0.11%	556
GAA	E	1.37	0.14%	1,335	CGA	R	1.05	0.11%	534
GAG	E	0.63	0.06%	621	CGC	R	0.44	0.04%	226
UUC	F	0.73	0.07%	1,350	CGG	R	0.70	0.07%	354
UUU	F	1.27	0.13%	2,366	CGU	R	0.74	0.07%	374
GGA	G	1.47	0.15%	801	AGC	S	0.56	0.06%	426
GGC	G	0.56	0.06%	303	AGU	S	0.89	0.09%	668
GGG	G	0.95	0.10%	518	UCA	S	1.30	0.13%	977
GGU	G	1.02	0.10%	557	UCC	S	1.09	0.11%	824
CAC	H	0.56	0.06%	370	UCG	S	0.65	0.06%	488
CAU	H	1.44	0.14%	958	UCU	S	1.51	0.15%	1,136
AUA	I	1.03	0.10%	1,424	ACA	T	1.23	0.12%	655
AUC	I	0.69	0.07%	962	ACC	T	1.00	0.10%	534
AUU	I	1.28	0.13%	1,777	ACG	T	0.54	0.05%	284
AAA	K	1.40	0.14%	2,195	ACU	T	1.23	0.12%	654
AAG	K	0.60	0.06%	943	GUA	V	1.23	0.12%	718
CUA	L	0.93	0.09%	789	GUC	V	0.68	0.07%	393
CUC	L	0.74	0.07%	632	GUG	V	0.68	0.07%	398
CUG	L	0.52	0.05%	442	GUU	V	1.41	0.14%	819
CUU	L	1.23	0.12%	1,045	UGG	W	1.00	0.10%	687
UUA	L	1.43	0.14%	1,215	UAC	Y	0.62	0.06%	652
UUG	L	1.15	0.11%	972	UAU	Y	1.38	0.14%	1,450
AUG	M	1.00	0.10%	769	UAA	*	1.17	0.12%	1,163
AAC	N	0.63	0.06%	769	UAG	*	0.84	0.08%	831
AAU	N	1.37	0.14%	1,690	UGA	*	0.99	0.10%	980

Table S13: Codon usage and RSCU of *I. faberi* chloroplast genome.

Codon	AA	RSCU	% of AA	Count	Codon	AA	RSCU	% of AA	Count
GCA	A	1.21	0.93%	470	CCA	P	1.28	1.53%	777
GCC	A	0.88	0.68%	343	CCC	P	0.99	1.18%	600
GCG	A	0.60	0.46%	232	CCG	P	0.62	0.74%	374
GCT	A	1.30	1.00%	506	CCT	P	1.10	1.32%	669
TGC	C	0.79	0.83%	422	CAA	Q	1.39	1.98%	1,006
TGT	C	1.21	1.28%	651	CAG	Q	0.61	0.86%	438
GAC	D	0.59	0.88%	447	AGA	R	2.05	2.06%	1,043
GAT	D	1.41	2.09%	1,061	AGG	R	1.18	1.19%	601
GAA	E	1.38	2.57%	1,304	CGA	R	0.95	0.96%	486
GAG	E	0.62	1.15%	583	CGC	R	0.48	0.49%	246
TTC	F	0.76	2.81%	1,424	CGG	R	0.69	0.70%	353
TTT	F	1.24	4.62%	2,341	CGT	R	0.64	0.65%	328
GGA	G	1.40	1.48%	753	AGC	S	0.57	0.86%	437
GGC	G	0.57	0.61%	307	AGT	S	0.86	1.31%	663
GGG	G	1.06	1.12%	567	TCA	S	1.30	1.96%	996
GGT	G	0.97	1.03%	520	TCC	S	1.14	1.73%	875
CAC	H	0.56	0.70%	353	TCG	S	0.66	1.00%	508
CAT	H	1.44	1.79%	909	TCT	S	1.47	2.23%	1,131
ATA	I	1.00	2.80%	1,418	ACA	T	1.20	1.22%	619
ATC	I	0.71	1.97%	1,001	ACC	T	0.99	1.01%	510
ATT	I	1.29	3.60%	1,823	ACG	T	0.57	0.58%	292
AAA	K	1.37	4.38%	2,221	ACT	T	1.24	1.27%	642
AAG	K	0.63	2.02%	1,022	GTA	V	1.20	1.34%	682
CTA	L	0.96	1.60%	813	GTC	V	0.74	0.83%	423
CTC	L	0.80	1.35%	683	GTG	V	0.67	0.75%	379
CTG	L	0.54	0.90%	458	GTT	V	1.39	1.56%	790
CTT	L	1.16	1.95%	989	TGG	W	1.00	1.35%	683
TTA	L	1.28	2.14%	1,086	TAC	Y	0.63	1.25%	633
TTG	L	1.25	2.10%	1,063	TAT	Y	1.37	2.73%	1,385
ATG	M	1.00	1.65%	836	TAA	*	1.24	2.44%	1,237
AAC	N	0.61	1.52%	770	TAG	*	0.77	1.52%	770
AAT	N	1.39	3.48%	1,766	TGA	*	0.99	1.96%	992

Table S14: Codon usage and RSCU of *I. oxyanthera* chloroplast genome.

Codon	AA	RSCU	% of AA	Count	Codon	AA	RSCU	% of AA	Count
GCA	A	1.09	0.78%	396	CCA	P	1.28	1.43%	723
GCC	A	0.97	0.69%	351	CCC	P	1.02	1.13%	573
GCG	A	0.63	0.45%	228	CCG	P	0.63	0.70%	354
GCT	A	1.31	0.94%	475	CCT	P	1.07	1.19%	602
TGC	C	0.81	0.89%	451	CAA	Q	1.42	2.05%	1,035
TGT	C	1.19	1.32%	669	CAG	Q	0.58	0.84%	426
GAC	D	0.53	0.76%	384	AGA	R	2.08	2.11%	1,068
GAT	D	1.47	2.13%	1,078	AGG	R	1.21	1.22%	619
GAA	E	1.38	2.55%	1,288	CGA	R	0.94	0.96%	484
GAG	E	0.62	1.15%	581	CGC	R	0.51	0.52%	263
TTC	F	0.76	2.79%	1,412	CGG	R	0.60	0.61%	310
TTT	F	1.24	4.57%	2,312	CGT	R	0.67	0.68%	342
GGA	G	1.37	1.45%	732	AGC	S	0.67	1.05%	532
GGC	G	0.64	0.68%	344	AGT	S	0.90	1.41%	715
GGG	G	0.98	1.03%	523	TCA	S	1.15	1.81%	917
GGT	G	1.00	1.06%	537	TCC	S	1.15	1.81%	917
CAC	H	0.57	0.76%	382	TCG	S	0.68	1.07%	540
CAT	H	1.43	1.90%	961	TCT	S	1.45	2.27%	1,147
ATA	I	1.03	2.81%	1,422	ACA	T	1.13	1.26%	639
ATC	I	0.74	2.04%	1,032	ACC	T	1.13	1.27%	642
ATT	I	1.23	3.37%	1,704	ACG	T	0.60	0.68%	342
AAA	K	1.40	4.31%	2,179	ACT	T	1.13	1.27%	642
AAG	K	0.60	1.84%	932	GTA	V	1.12	1.18%	598
CTA	L	0.79	1.29%	654	GTC	V	0.73	0.77%	390
CTC	L	0.73	1.19%	603	GTG	V	0.73	0.77%	390
CTG	L	0.57	0.93%	468	GTT	V	1.41	1.49%	752
CTT	L	1.25	2.05%	1,035	TGG	W	1.00	1.39%	703
TTA	L	1.36	2.21%	1,119	TAC	Y	0.67	1.42%	717
TTG	L	1.29	2.10%	1,063	TAT	Y	1.33	2.82%	1,426
ATG	M	1.00	1.54%	776	TAA	*	1.22	2.52%	1,275
AAC	N	0.59	1.48%	746	TAG	*	0.83	1.71%	865
AAT	N	1.41	3.50%	1,768	TGA	*	0.95	1.97%	998

Table S15: Codon usage and RSCU of *I. tienchuanensis* chloroplast genome.

Codon	AA	RSCU	% of AA	Count	Codon	AA	RSCU	% of AA	Count
GCA	A	1.15	0.79%	399	CCA	P	1.31	1.47%	744
GCC	A	0.96	0.66%	333	CCC	P	1.02	1.14%	577
GCG	A	0.66	0.45%	230	CCG	P	0.64	0.72%	366
GCT	A	1.23	0.84%	426	CCT	P	1.03	1.16%	588
TGC	C	0.81	0.93%	469	CAA	Q	1.39	2.11%	1,071
TGT	C	1.19	1.36%	689	CAG	Q	0.61	0.93%	469
GAC	D	0.58	0.84%	424	AGA	R	2.06	2.07%	1,051
GAT	D	1.42	2.05%	1,038	AGG	R	1.19	1.20%	608
GAA	E	1.38	2.67%	1,355	CGA	R	0.89	0.90%	455
GAG	E	0.62	1.20%	609	CGC	R	0.49	0.49%	247
TTC	F	0.76	2.85%	1,444	CGG	R	0.71	0.71%	361
TTT	F	1.24	4.70%	2,379	CGT	R	0.67	0.68%	344
GGA	G	1.41	1.43%	726	AGC	S	0.66	1.01%	514
GGC	G	0.63	0.64%	324	AGT	S	0.83	1.28%	651
GGG	G	1.00	1.02%	518	TCA	S	1.27	1.95%	986
GGT	G	0.96	0.98%	497	TCC	S	1.15	1.76%	891
CAC	H	0.58	0.79%	402	TCG	S	0.65	1.01%	511
CAT	H	1.42	1.92%	975	TCT	S	1.44	2.21%	1,122
ATA	I	1.04	2.79%	1,416	ACA	T	1.15	1.21%	611
ATC	I	0.72	1.94%	981	ACC	T	1.06	1.11%	564
ATT	I	1.25	3.37%	1,710	ACG	T	0.65	0.68%	346
AAA	K	1.36	4.16%	2,108	ACT	T	1.13	1.18%	598
AAG	K	0.64	1.96%	995	GTA	V	1.18	1.25%	632
CTA	L	0.97	1.64%	833	GTC	V	0.78	0.83%	419
CTC	L	0.74	1.27%	642	GTG	V	0.69	0.72%	367
CTG	L	0.53	0.90%	455	GTT	V	1.35	1.42%	720
CTT	L	1.18	2.02%	1,024	TGG	W	1.00	1.41%	715
TTA	L	1.35	2.30%	1,166	TAC	Y	0.66	1.36%	690
TTG	L	1.23	2.10%	1,065	TAT	Y	1.34	2.74%	1,388
ATG	M	1.00	1.52%	768	TAA	*	1.22	2.55%	1,293
AAC	N	0.59	1.45%	733	TAG	*	0.81	1.69%	858
AAT	N	1.41	3.43%	1,739	TGA	*	0.98	2.05%	1,039

Table S16: Codon usage and RSCU of *I. blepharosepala* chloroplast genome.

Codon	AA	RSCU	% of AA	Count	Codon	AA	RSCU	% of AA	Count
GCA	A	1.15	0.90%	458	CCA	P	1.29	1.49%	756
GCC	A	0.91	0.72%	364	CCC	P	1.02	1.17%	595
GCG	A	0.56	0.44%	222	CCG	P	0.58	0.67%	342
GCT	A	1.38	1.09%	553	CCT	P	1.11	1.28%	651
TGC	C	0.77	0.88%	446	CAA	Q	1.44	2.03%	1,032
TGT	C	1.23	1.40%	709	CAG	Q	0.56	0.80%	406
GAC	D	0.53	0.78%	396	AGA	R	1.94	2.06%	1,044
GAT	D	1.47	2.17%	1,103	AGG	R	1.10	1.17%	595
GAA	E	1.37	2.61%	1,327	CGA	R	1.04	1.10%	560
GAG	E	0.63	1.19%	606	CGC	R	0.49	0.51%	261
TTC	F	0.72	2.65%	1,347	CGG	R	0.67	0.71%	361
TTT	F	1.28	4.71%	2,390	CGT	R	0.77	0.81%	413
GGA	G	1.44	1.51%	767	AGC	S	0.56	0.87%	444
GGC	G	0.58	0.61%	309	AGT	S	0.89	1.38%	701
GGG	G	0.98	1.02%	518	TCA	S	1.29	2.00%	1,018
GGT	G	1.00	1.04%	529	TCC	S	1.15	1.78%	904
CAC	H	0.60	0.75%	382	TCG	S	0.64	0.98%	500
CAT	H	1.40	1.77%	898	TCT	S	1.48	2.29%	1,165
ATA	I	1.04	2.85%	1,448	ACA	T	1.22	1.32%	672
ATC	I	0.68	1.86%	943	ACC	T	0.98	1.06%	536
ATT	I	1.28	3.53%	1,792	ACG	T	0.59	0.64%	324
AAA	K	1.42	4.37%	2,221	ACT	T	1.21	1.31%	663
AAG	K	0.58	1.80%	913	GTA	V	1.20	1.26%	640
CTA	L	0.91	1.49%	759	GTC	V	0.74	0.78%	398
CTC	L	0.70	1.15%	584	GTG	V	0.68	0.71%	362
CTG	L	0.54	0.88%	449	GTT	V	1.39	1.47%	744
CTT	L	1.26	2.07%	1,049	TGG	W	1.00	1.35%	684
TTA	L	1.39	2.28%	1,156	TAC	Y	0.62	1.22%	621
TTG	L	1.21	1.98%	1,006	TAT	Y	1.38	2.70%	1,372
ATG	M	1.00	1.56%	793	TAA	*	1.20	2.41%	1,225
AAC	N	0.61	1.51%	765	TAG	*	0.81	1.62%	825
AAT	N	1.39	3.45%	1,754	TGA	*	0.99	1.99%	1,011

Table S17: Codon usage and RSCU of *I. distracta* chloroplast genome.

Codon	AA	RSCU	% of AA	Count	Codon	AA	RSCU	% of AA	Count
GCA	A	1.08	0.82%	415	CCA	P	1.28	1.50%	762
GCC	A	0.96	0.73%	369	CCC	P	0.95	1.12%	567
GCG	A	0.60	0.46%	231	CCG	P	0.59	0.70%	353
GCT	A	1.35	1.02%	517	CCT	P	1.19	1.40%	710
TGC	C	0.78	0.79%	403	CAA	Q	1.40	2.09%	1,059
TGT	C	1.22	1.24%	627	CAG	Q	0.60	0.90%	455
GAC	D	0.53	0.79%	401	AGA	R	1.96	2.02%	1,025
GAT	D	1.47	2.18%	1,105	AGG	R	1.18	1.22%	618
GAA	E	1.38	2.56%	1,298	CGA	R	1.04	1.07%	543
GAG	E	0.62	1.15%	585	CGC	R	0.47	0.49%	248
TTC	F	0.73	2.72%	1,377	CGG	R	0.67	0.69%	351
TTT	F	1.27	4.73%	2,397	CGT	R	0.69	0.71%	361
GGA	G	1.44	1.48%	750	AGC	S	0.65	0.96%	487
GGC	G	0.61	0.63%	317	AGT	S	0.94	1.38%	699
GGG	G	0.95	0.98%	495	TCA	S	1.19	1.76%	890
GGT	G	1.00	1.02%	519	TCC	S	1.16	1.71%	869
CAC	H	0.55	0.73%	372	TCG	S	0.70	1.03%	522
CAT	H	1.45	1.93%	977	TCT	S	1.37	2.03%	1,028
ATA	I	1.01	2.84%	1,439	ACA	T	1.14	1.19%	605
ATC	I	0.72	2.02%	1,023	ACC	T	1.02	1.07%	544
ATT	I	1.27	3.55%	1,802	ACG	T	0.59	0.62%	312
AAA	K	1.36	4.34%	2,202	ACT	T	1.26	1.32%	668
AAG	K	0.64	2.06%	1,044	GTA	V	1.17	1.31%	663
CTA	L	0.90	1.51%	765	GTC	V	0.73	0.82%	414
CTC	L	0.73	1.23%	624	GTG	V	0.68	0.75%	382
CTG	L	0.56	0.94%	476	GTT	V	1.42	1.59%	807
CTT	L	1.28	2.14%	1,085	TGG	W	1.00	1.41%	713
TTA	L	1.27	2.12%	1,076	TAC	Y	0.61	1.24%	630
TTG	L	1.26	2.11%	1,071	TAT	Y	1.39	2.83%	1,433
ATG	M	1.00	1.57%	796	TAA	*	1.22	2.29%	1,163
AAC	N	0.59	1.48%	749	TAG	*	0.83	1.56%	790
AAT	N	1.41	3.56%	1,805	TGA	*	0.96	1.82%	921

Table S18: Sampling information for 9 *Impatiens* plants

**Ordinal number**	**Latin name**	**Sampling location**	**Distribution altitude/m**	**Habitat profile**
1	*I. repens*	Sri Lanka	1686-1697	Roadside
2	*I. rectirostrata*	Dujiangyan, Chengdu	1318-1332	Forest edge, roadside
3	*I. baishaensis*	Jinkouhe District	1639-1661	Alongside road ditches
4	*I. rostellata*	Hongya County, Sichuan Province	1607-1621	Roadside
5	*I. faberi*	Hongya County, Sichuan Province	1500-1534	Roadside ditches
6	*I. oxyanthera*	Baoxing County, Sichuan Province	1276-1392	Roadside
7	*I. tienchuanensis*	Emei Mountain in Sichuan Province	1232-1272	Streamside, roadside
8	*I. blepharosepala*	Hongya County, Sichuan Province	1500-1539	Cliffside, ditchside
9	*I. distracta*	Hongya County, Sichuan Province	1452-1473	Roadside

Table S19: GenBank numbers of 9 *Impatiens* plants

**Ordinal number**	**Latin name**	**Genbank number**
1	*I. repens*	PQ649850
2	*I. rectirostrata*	PQ647017
3	*I. baishaensis*	PQ647014
4	*I. rostellata*	PQ647018
5	*I. faberi*	PQ652174
6	*I. oxyanthera*	PQ647016
7	*I. tienchuanensis*	PQ647019
8	*I. blepharosepala*	PQ643224
9	*I. distracta*	PQ647015
